# Forecasting Africa’s fertility decline by female education groups

**DOI:** 10.1073/pnas.2320247121

**Published:** 2024-11-04

**Authors:** Saroja Adhikari, Wolfgang Lutz, Endale Kebede

**Affiliations:** ^a^Kinship Inequalities Group, Max Planck Institute for Demographic Research, Rostock 18057, Germany; ^b^Asian Demographic Research Institute, Shanghai University, Shanghai 200444, China; ^c^Wittgenstein Centre for Demography and Global Human Capital, International Institute for Applied Systems Analysis (IIASA), Austrian Academy of Sciences, University of Vienna, Vienna 1010, Austria; ^d^Department of Demography, University of Vienna, Vienna 1010, Austria

**Keywords:** forecasting, cohort fertility, Africa, female education, skills

## Abstract

While female education has long been recognized as a key driver of fertility decline during the process of demographic transition and most population projection models consider it implicitly or explicitly in their forecasts of overall fertility, there still is need for a method to forecast education-specific fertility trends directly. Here we propose a method for projecting education-specific fertility declines for cohorts of women in Sub-Saharan Africa based on all available demographic and health surveys data for African countries (including 1.03Mio cases). We study at different levels of aggregation (sample clusters, strata, and national) the associations between ideal family size and completed cohort fertility for education groups, on the one hand, and the average level of education in those units, on the other. The consistently very strong empirical associations suggest a plausible narrative by which a higher prevalence of educated women in a spatial unit influences the fertility levels of women in all specific education categories. Empirical associations between education-specific cohort fertility trends at the national level and newly available quality-adjusted human capital data for these cohorts are then operationalized to produce education-specific population projections as they are needed for—among other uses—the shared socioeconomic pathways scenarios that are widely used in the climate change research community. Sensitivity analyses including out-of-sample projections support the validity of the proposed method which is then applied to 37 African countries.

## Significance

Africa’s population is expected to more than double before 2100. It still has the world’s highest fertility rates, and the future of world population growth will depend on the speed of fertility decline in Africa. Demographic trends also matter for health and future economic development as well as for interactions with the natural environment where population scenarios by age, sex, and level of education are now widely used outside the demographic community for assessing the mitigative and adaptive capacity to climate change. But a method for forecasting education-specific fertility trends has still been lacking. Here, we propose such a method, also strengthening the case for policies enhancing female education and thus accelerating the fertility transition in Africa.

The world is currently going through a fundamental demographic transition from high and initially largely uncontrolled birth and death rates to low and controlled birth and death rates. Different populations are at different stages of this universal process that started in Northern Europe and France in the 19th Century and is still unfolding in most of Africa and parts of Western Asia. Typically, death rates began to decline before birth rates because people were ready to quickly move to newly available health interventions that helped them or their family members to survive, whereas reproductive behavior has been much more deeply ingrained in traditional norms, cultural values and transmitted behavioral patterns and thus changes more slowly. Sub-Saharan Africa is currently still in the midst of this fertility transition, and due to the increasing proportion of Africa in the world population, the future speed of fertility decline in Africa will be a key determinant of future world population growth.

Population projections are used for a wide array of applications in social and economic planning. Recently, they have also found prominent applications in the field of climate change analyses, both with respect to options for reducing greenhouse gas emissions (mitigation) as well as for forecasting societies’ adaptive capacities to already unavoidable climate change. Population projections have long been an integral part of the scenarios used in the international climate change research community that have informed the series of Intergovernmental Panel on Climate Change (IPCC) reports. While for earlier reports the SRES (1) only considered alternative future pathways of total population size—with population mostly serving the function as a denominator for per capita indicators—the currently used Shard Socioeconomic Pathways (SSP) scenarios cross-classify populations not only by the conventional age and sex structures but also by six levels of educational attainment. And these scenarios on different human capital trajectories are deeply interwoven with other economic, social, and technological dimensions of the SSPs (2, 3). In particular, human capital is considered a key driver of economic growth and of enhancing the adaptive capacity of societies to increasing hazards associated with climate change.

These multidimensional models of population dynamics stratifying populations by age, sex, level of education, and in some cases also labor force participation and other demographic dimensions built on the methods of multistate demography as developed by refs. 4, 5 and have over the years been further validated and applied to all countries of the world (6–8).This multidimensional approach offers two important advantages over the conventional cohort-component approach by age and sex alone: a) it explicitly incorporates further important sources of heterogeneity such as level of education—in virtually all societies fertility, mortality, and migration show marked differences by level of education—and thus results in different forecasts of population size and age structure, depending on the future of education (9); b) It provides more relevant demographic information for the users of population projections such as human capital forecasts that are key determinants of many social and economic aspects. This is also the reason why the climate change and integrated assessment modeling community chose to use these multidimensional projections, rather than conventional projections by age and sex alone (3).

One limitation for the universal application of population projections which are fully stratified by level of education in addition to age and sex is the availability of time-series data on vital rates stratified by level of education. This is particularly relevant in the case of future fertility trends for different female education groups where currently assumptions are essentially based on the forecast of overall fertility trends which then in a second step—based on additional assumptions of proportionality—are broken down into education-specific fertility trends (10). Since this is unsatisfactory from a theoretical point of view and recently new estimates of education- and age-specific fertility rates for a large number of countries have become available (11), this paper proposes a method for directly forecasting education-specific fertility rates without having to go via the assumption of overall fertility rates. The proposed approach is in accordance with the widely discussed and partially controversial diffusion theory (12) but does not depend on its acceptance as the main causal driver of the process as will be discussed in the section on functional causality below.

The paper is structured in the following way. After a literature review on diffusion processes in the field of reproductive ideals and behavior, the role of education in the concept of cognition-driven demographic transition and discussions of functional causality in the context of fertility projections, the paper investigates empirical associations using the full set of 138 DHS that have been carried out in Sub-Saharan Africa since the 1986, including 1.03 Mio individual records of ever married women aged 15 to 49. We group women into four different categories of educational attainment and study their education-specific ideal family size as well as completed cohort fertility at the level of 58,708 sample clusters and 11,873 strata relating this to the mean level of education in the respective cluster/stratum, thus empirically assessing the degree of social embeddedness of reproductive behavior under a cross-sectional perspective.

In the following part of the paper, we move to a longitudinal perspective, where the level of analysis is limited to the national level and we convert the recently available age- and education-specific fertility rates for most African countries into education-specific cohort fertility trends to avoid tempo distortions and have more stable trends for applying and testing the forecasting model proposed. After estimating completed cohort fertility for some of the younger cohorts in order to generate longer time-series, we then conduct extensive out-of-sample projection exercises which demonstrate high accuracy, particularly when using new estimates of Skills in Literacy Adjusted Mean Years of Schooling (SLAMYS) rather than the conventional MYS as aggregate human capital indicator, thus incorporating the quality of education. Finally, this method based on SLAMYS is used to project education-specific fertility for future cohorts and the findings as well as remaining research needs are critically discussed.

## Female Education, Demographic Transition, and Possible Diffusion Processes

While the concept of demographic transition is almost a century old (13, 14), there still is no consensus about what drives this universal process. In the most general way, it has been attributed to “demographic modernization,” a term that avoids a more specific identification of the determinants. With respect to mortality decline, the differences in opinion mostly concern the relative role of nutrition and economic growth on the one hand and medical progress on the other. With respect to fertility, there have been essentially three broad explanations competing with each other: ideational/cognitive change, socioeconomic change, and family planning/reproductive health programs (7). But in fact, these three explanations are not necessarily in competition with each other because they operate at different levels and jointly result in a lasting fertility decline.

This view directly corresponds to the famous three prerequisites of a lasting fertility decline as identified by Ansley Coale in 1973 (15) on the basis of insights from the Princeton European Fertility Project. The first of these stated prerequisites is that “fertility needs to be within the calculus of conscious choice”, which has been further elaborated by Van de Walle (16) under the title “Fertility Transition, Conscious Choice and Numeracy.” Cleland et al. (17) also point to ideational changes as the trigger of the fertility transition rather than changing economic conditions or contraceptive methods. These latter two levels of determination are captured by Coale’s two other stated preconditions, namely that lower fertility needs to be advantageous and that acceptable means for family limitation need to be available. Again, these three preconditions operate at different levels and need to be simultaneously met to result in a lasting fertility decline. But there still is discussion about which of these levels trigger changes in the other ones.

It has recently been argued under the heading of “cognition-driven demographic transition” that the spread of literacy and associated cognitive and abstraction skills have been the decisive triggers of the subsequent medical/technological, social, and economic changes that together resulted in the mortality and fertility transitions (7, 18). This concept is fully in line with the influential economic “unified growth theory” by Galor (19) that views the industrial revolution and modern economic growth as being initiated by the spread of literacy and investments in human capital. And the spread in literacy, starting from Northern Europe has in turn been linked to the protestant reformation as a noneconomic ideational trigger of the subsequent global transformations (20, 21). While the literature on literacy and cognition as a trigger of modern economic growth and the global demographic transition cannot be summarized here, the timing of its global diffusion is of direct relevance to this paper. The timing of the spread of universal literacy—importantly including female literacy—from Northern Europe and France to Southern Europe, the Americas, Eastern Asia—with Japan spearheading the process followed by extremely rapid education expansions in South Korea and China—followed by Western Asia, with Sub-Saharan Africa being the last to enter this process, closely resembles the timing of demographic transitions in different parts of the world. And even within Africa marked regional differences appear, with Northern and Southern Africa being more advanced both in terms of female literacy/education as well as fertility decline and generally Eastern Africa being somewhat more advanced than Western Africa in both processes.

An extensive body of research has established a strong link between higher female education and lower fertility which is particularly pronounced in all societies that are still in the process of demographic transition. Many studies have also supported the assumption of causality in the determination of fertility through the analysis of natural experiments or other methods, which is summarized in ref. 22. Despite this robust evidence for a causal effect of female education on fertility, it is still less clear which aspects of education cause these changes. Education tends to among other things lead to higher incomes and higher labor force participation—thus increasing the opportunity costs of having children—as well as to generally higher socioeconomic status. In developmental psychology, it has also been shown beyond any doubt that quality education alters the way we perceive the world, changes our planning horizon, and the degree to which we think abstractly and can imagine counterfactual scenarios, all things that matter for our choices and our behavior (23–26). Hence, in the context of demographic transition, we should focus on the quality of education and the skills—including abstraction skills and skills of independent judgment—rather than just formal educational attainment or the years a girl spent in school irrespective of the learning outcomes. Unfortunately, direct measurements of skills and cognition of adult populations in developing countries are still very limited. At the macrolevel of the working-age populations in different countries global estimates of Skills in Literacy Adjusted Mean Years of Schooling (SLAMYS) have recently become available (27). But for studies at the microlevel, most surveys only provide information on years spent in school or highest educational attainment. Since there still is a high correlation at the individual level between years spent in school and tested skills, formal education is widely used as a proxy for skills and cognition. In this paper, we will thus use formal education as a proxy for skills when we refer to individual data or education groups as derived from DHS, but use the newly available SLAMYS data for measuring quality-adjusted education for the analysis of national trends that drive the national-level fertility projections.

One of the possible mechanisms by which fertility changes within subgroups of a population is diffusion, which is a process by which the views and behaviors of people are influenced by the actions and attitudes of others within their society. Human beings are inherently social creatures and individuals tend to seek conformity or validation for their behavior from their peers, family, and community members (28). The collective behavior that emerges from these interactions is often regarded as social norms, which serve as guidelines for acceptable conduct within a given society (29). In the field of demography there is a long tradition of exploring the impact of social interactions on reproductive decisions, with most studies in the filed having primarily focused on contraception and its acceptance through social learning (30–32) or qualitatively looked at the stated social influence on fertility intentions (33). Less attention has been given to quantitatively capturing such possible social learning processes in the idealization and behavior surrounding human reproduction at the individual level (34). In 2001, the National Research Council published an assessment, “Diffusion Processes and the Fertility Transition,” summarizing the literature and pointing out that most of the studies in the field focus more on how diffusion occurs rather than what actually diffuses (12).

The question of what is being disseminated also depends on what is being studied and measured as outcome variables of assumed diffusion processes. This is also directly related to the tricky question of how to assess the causality in such processes. Are we only observing correlations or is there reason to assume that in fact one individual influences the others in their behaviors? The literature on this identification of endogenous social effects has been mostly inconclusive. Ref. 35 suggests as a promising route for resolving the issue, to go beyond empirically observed behavioral data and collect additional information either through experiments or through additional subjective data such as those relating to intentions. Since, for the time being, it is not possible to conduct experiments on this issue in Africa, we follow the second strategy and take advantage of the rich data on intended or ideal family size and actual live births as collected by DHS. For this reason, our paper starts with a focus on ideal family size within different education groups at the level of sample clusters and the somewhat larger strata in which interactions and social learning can be assumed to be operating. This initial focus on ideal family size has the advantage of also directly relating to the theoretical framework of ideational change as a driver of the fertility transition. One possible mechanism could thus be that lower fertility ideals and in consequence lower actual fertility emerges among higher educated women who are ahead of others in recognizing the advantages of having a smaller family. This would be in line Bongaarts and Watkins (34), who suggested that apart from socioeconomic determinants, fertility declines are accelerated by social interaction in close geographical or social proximity. Through this mechanism, lower fertility ideals and subsequent behaviors would then diffuse to less-educated women living close to more-educated women. In our analysis, we further differentiate the pattern by not just looking at two groups but at four different groups of women as defined by their educational attainment since we have large international datasets allowing for such differentiation.

## Patterns of Ideal Number of Children and Completed Cohort Fertility From DHS

**Fig. 1.**
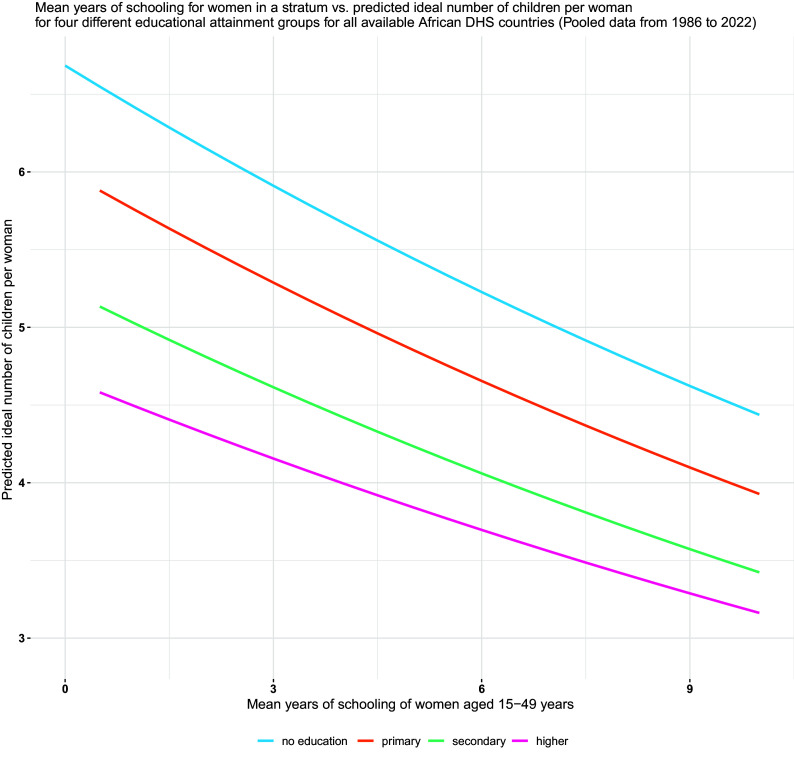
Mean years of schooling for women in a stratum vs. predicted ideal number of children per woman for four different educational attainment groups for all available African DHS countries.

Fig. 1 shows on the right-hand side the estimated relationship between ideal family size as stated by the 1.03 million ever-married women aged 15 to 49 in all African DHS surveys pooled together and differentiated by the highest educational attainment category of the woman (no education, primary, secondary, and higher) and plotted against the average level of education (Mean Years of Schooling) of all women in the same sample stratum (group of several smaller clusters). There are 11,873 such strata in this dataset. This pattern emerges from the estimates of a fixed-effect Poisson regression model as described in the methods section below.

The result shows the expected apparent differentiation of ideal family size by level of education and a less expected very strong decline within each education category with increasing average education in the sample stratum as plotted on the *x*-axis. The registered average ideal family size for women without formal education varies from around seven children in strata with very few educated women to only around 4.5 children in those with high average education, as captured by high mean years of schooling. For women with primary and secondary education, the lines are lower but have a similar slope. This strong effect of the presence of more-educated women with lower fertility preferences in the same living environment on the stated preferences of women with a given individual education is consistent with the assumption of strong diffusion effects in fertility preferences. Viewed differently, the strong decline of, e.g., the green line indicates that the ideal family size of women with secondary education in an otherwise uneducated environment turns out to be about 50 percent higher than in a highly educated environment.

This finding indicates a strong and regular empirical association in the expected direction as it would be consistent with a possible diffusion effect. It does not, however, prove a true causal effect, since this pattern could also result from other factors that are not covered in this model. But this difficulty in demonstrating strong causality with the given data and in the absence of experimental designs need not stop us from trying to use this pattern for forecasting based on the concept of functional causality (36) as it is often applied in demographic forecasting and has been operationalized the field of forecasting the relationship between female education and fertility (22). It requires three preconditions to be met: a) a strong empirical association between the factors studied, b) a plausible narrative about the mechanisms through which one influences the other and c) ruling out other obvious competing explanations such as reverse causality c.1) or joint determination by a third factor c.2). If these conditions are met, it is plausible to have the working assumption of a continuation of the observed relationship over the projection horizon and for the populations under consideration.

In assessing these three preconditions, the strong empirical association is evident from the pattern described in Fig. 1 above as well as from the further figures included in *SI Appendix*. The plausible narrative of influence under which a more innovative avantgarde (the more-educated women) serves as a role model for the less-educated segments of the population through social interactions is well described in the extensive literature on social diffusion (12, 34) which was summarized in the previous section and fits well with the empirical findings presented in this study. The third condition of addressing possible alternative mechanisms of determination is the most difficult to demonstrate. The possibility of reverse causation c.1) from a higher ideal family size to lower education does not have a plausible mechanism due to the fact that in the typical life course education comes before the age at which women are included in the sample. The possible joint determination by a third factor c.2) such as a more conservative environment leading jointly to lower female education and higher fertility ideals is more difficult to rule out. We address this issue through various sensitivity analyses as documented in *SI Appendix* with a specific focus on the level of urbanization which tends to be associated with both better access to education and lower fertility ideals. Since urban/rural place of residence is also included in the DHS microdata, in our sensitivity runs we also included it as an additional predictor variable in the model. The results as presented in *SI Appendix* show that still almost identical associations appear once distinguishing between rural and urban strata. While this seems to rule out an obvious competing mechanism, there could still be other third factors such as government policies, influence by media or local traditions and ethnicity, which we tried to address—for lack of more specific data—through assessing the relationship at very different levels of spatial aggregation ranging from 58,708 small sample clusters to the 11,873 strata to national to pan-African level. At all these levels essentially, the same patterns appear with the expected difference that the smaller the spatial unit the stronger the associating thus likely reflecting a higher degree of interaction and diffusion. Given these sensitivity analyses, it seems justified to assume functional causality at least for the projection horizon and the countries under consideration. But, again, the presence of strong diffusion processes is not a necessary precondition for applying the proposed forecasting method. The method would also work, if some of these other factors were involved in generating the observed pattern as long as the nature of the pattern of determination does not change fundamentally over the chosen time horizon. But still it makes sense to view diffusion processes as prime candidate for a plausible narrative behind the method.

## Operationalization for Projections of Education-Specific Cohort Fertility

While the narrative for ideational diffusion rests primarily on expressed ideal family size, in our projection model for forecasting education-specific fertility we cannot rely on data for ideal family size since this is only available in sample surveys. Since the projection model will rely on completed cohort fertility at the national level, we will as a next step study the relationships between actual fertility and education first at the level of DHS clusters and then on national level.

**Fig. 2.**
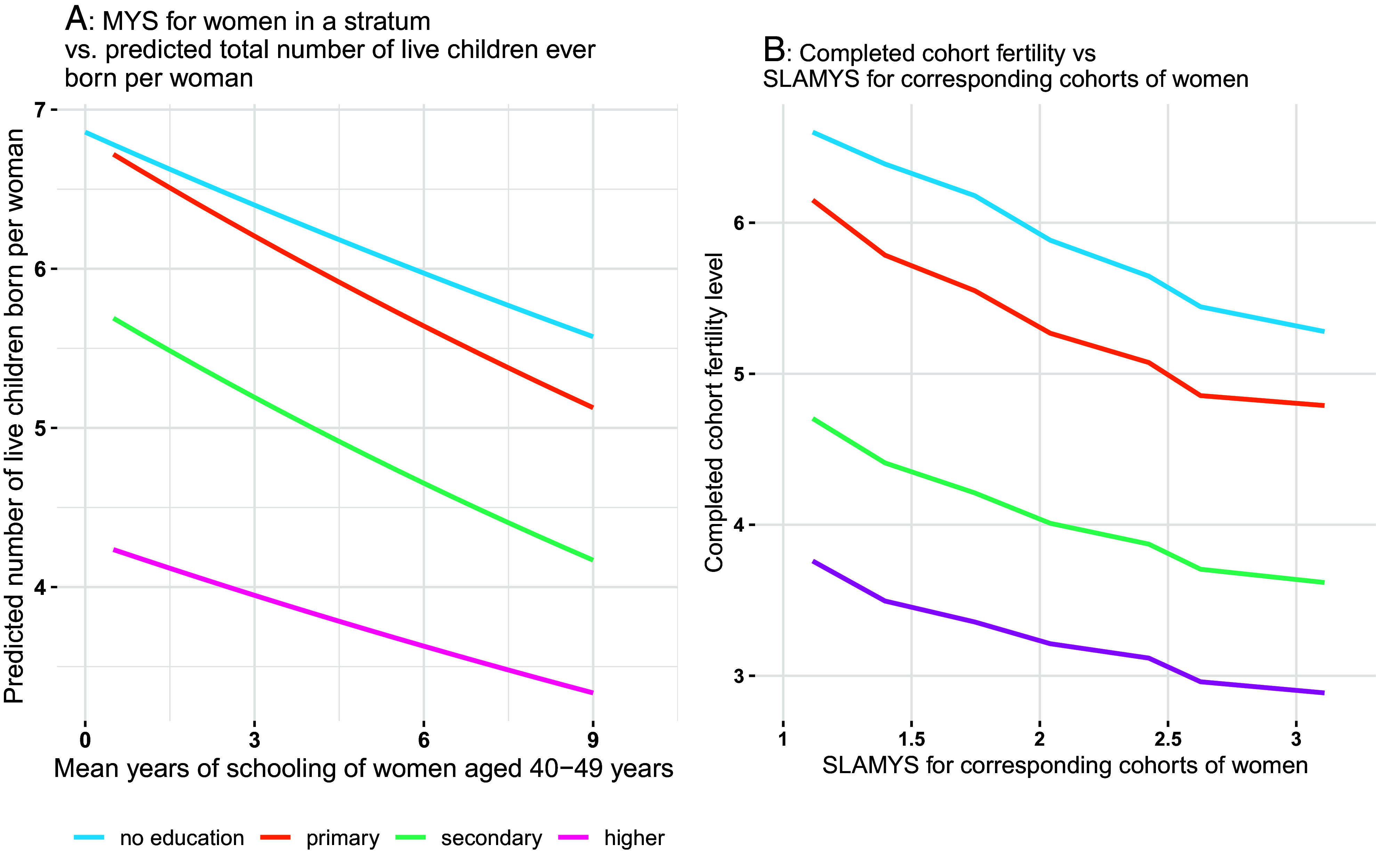
(*A*) Mean years of schooling for women in a stratum vs. predicted total number of live children ever born per woman for four different educational attainment groups for all available African DHS countries. (*B*) Completed cohort fertility (national level data combined for Africa) on the vertical axis against SLAMYS for corresponding cohorts of women.

The results shown in Fig. 2*A* are isomorphic to those in Fig. 1 but with completed cohort fertility from DHS (approximated by live children ever born of the age group 40 to 49) instead of ideal fertility on the vertical axis and mean years of schooling of all women in the relevant cohorts on the horizontal axis (assuming that women mostly interact with others of roughly the same age). This indicates an even stronger differentiation by the level of women’s education, in particular for the highest education groups. In a low-educated environment, there seems to be little difference between the realized fertility of uneducated women and women with primary education. In contrast, women with secondary or higher education in such environments do already have significantly lower completed fertility. With increasing education in the environment, actual fertility in each education group becomes consistently lower. This suggests that the empirical pattern that is consistent with an assumed diffusion process also holds for actual completed cohort fertility in a similar way to ideal family size.

The above-described patterns derived from individual DHS data are based on cross-sections as well as pooled data of cross-sections at different points in time. For a fertility projection model at the national level, however, we rather want consistent time-series data at the national level which due to the irregular and often inconsistent pattern of DHS survey data cannot be derived directly form DHS. To deal with this problem, estimates of fully consistent national level time-series data for education- and age-specific period fertility rates for most African countries since 1970 have recently been published (11). Using Bayesian methods, this dataset uses information from all available DHS to estimate fully consistent (along cohort lines) education-specific time series in 5-y intervals of time and age that are also consistent with the UN estimates of overall fertility levels for 37 African countries. Since these period data of education- and age-specific fertility rates also exhibit some irregular temporal fluctuations—presumably in part due to tempo distortions—the forecasting model proposed here focuses on education-specific cohort fertility. For this purpose, the available time series 1970–2015 of period age-specific rates was converted into completed cohort fertility starting with the birth cohorts of 1955. In order to get a couple more data points, we estimated completed cohort fertility rates for the 1980–1984 and 1985–1989 birth cohorts—which otherwise would be truncated—using the Lee–Carter approach (37) as further developed by ref. 38. The resulting education-specific cohort fertility trends for all African countries combined are shown in Fig. 4. The data for individual countries as well as detailed description of the method are given in *SI Appendix*.

In a next step, these education-specific cohort fertility trends were related to the SLAMYS as discussed above to adjust for the quality dimension of education and have a better indicator of cognition rather than just years sitting in school. As described in *SI Appendix*, the SLAMYS were estimated as to be specific for the cohorts of women under consideration. The pattern appearing in Fig. 2*B* for African countries combined—as well as for virtually all individual countries—exhibits a clear and monotonous decline in education-specific cohort fertility as the education level in the environment (measured by SLAMYS) increases over time.

Based on this relationship we can calculate the average diffusion rates which are the basis for our proposed projection model. In a nutshell, the diffusion rate is defined as the average percentage change in education-specific cohort fertility as a consequence of a unit change in SLAMYS. The estimated average diffusion rates across Africa turn out to be −10.2%, −11.09%, −11.57%, and −11.64% for no education, primary education, secondary education, and higher education respectively. With given estimates and projections of the SLAMYS for cohorts up to the birth cohort of 2000, we then simply apply those estimated diffusion rates to the education-specific cohort fertility levels in the jump-off year of the projections. The results are shown for four examples of African countries in Fig. 4.

**Fig. 3.**
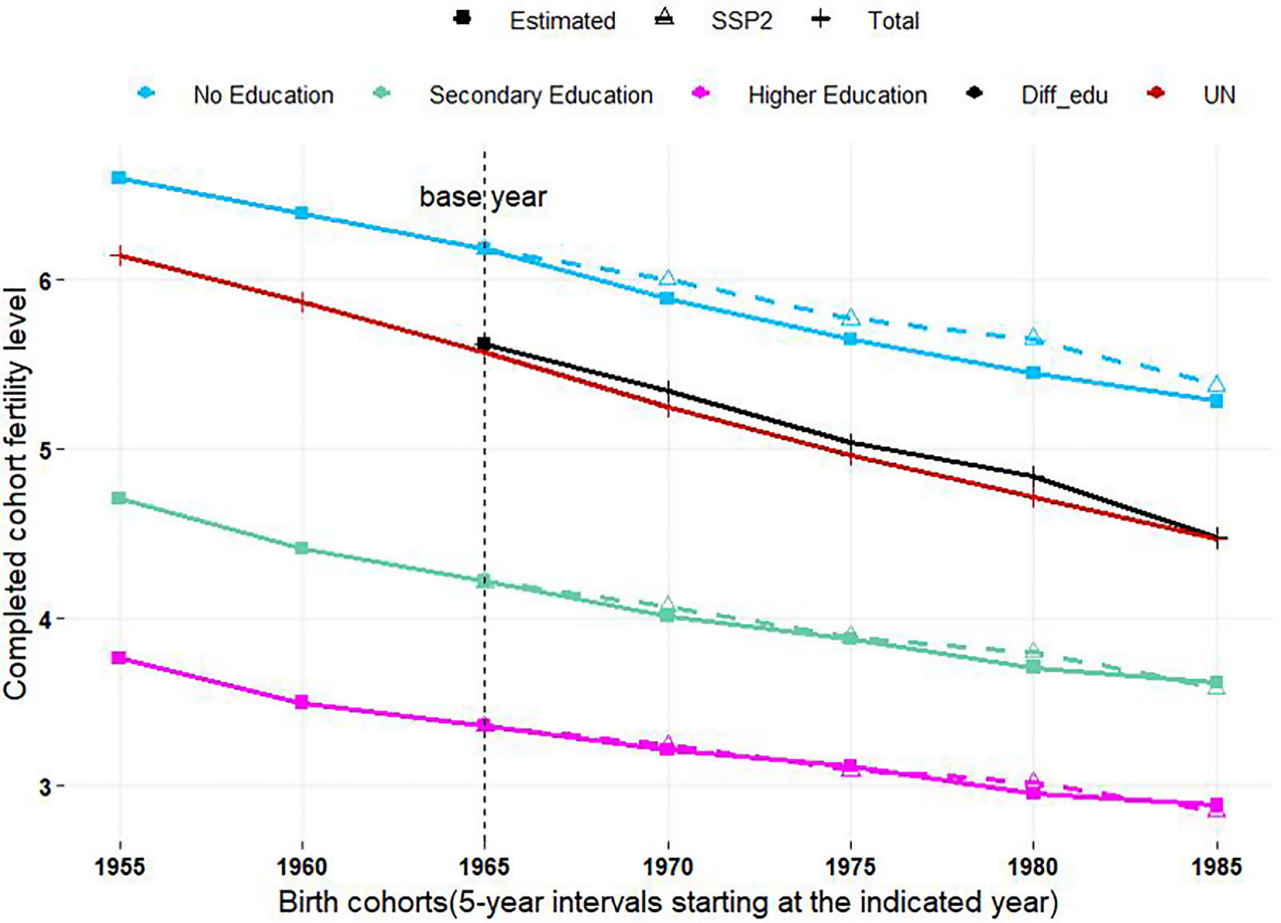
Out-of-sample projection of education-specific cohort fertility trends for African countries.

When a projection method is being proposed, it is a common procedure to test it through so-called out-of-sample projections, which implies in our case taking the empirical time-series of education-specific cohort fertility for only a subperiod and apply the method to project the series for the rest of the period for which empirical data are available. All together the empirical data include cohort fertility for seven 5-y birth cohorts from 1955–1959 to 1985–1989. Fig. 3 shows the results of such an exercise in which only the experience of the first three cohorts (up to the birth cohort 1965–1969) was taken as the empirical basis for the projections of the following four birth cohorts (up to 1985–1989). The figure shows the projected trends as dashed lines while the solid lines give the empirical trends for comparison. The results show an almost perfect prediction over time for the higher education groups and still a nice fit for uneducated women, particularly for the last birth cohort 1985–1989 while in the preceding cohorts, a minor overestimation of fertility can be observed. The solid black and red lines in the middle of the figure give the overall TFR trend resulting from aggregating the projected education-specific TFRs with applying cohort-specific education weights (black line) while the red line gives the empirical line which also corresponds to the UN data. This is again a very good fit, in particular, considering that base period was only three 5-year cohorts while the projection period was four such cohorts.

Fig. 3 “Out-of-sample” projection (dotted lines) of education-specific cohort fertility trends for African countries together with information of birth cohorts 1955–1959 to 1965–1969 taken as given and applying the projection method up to the one of 1985–1989, compared to the empirical trends (solid lines) for those cohorts. The solid black line shows the projected cohort fertility for all education groups combined as compared to the empirical data from the UN (solid red line). Because of overlapping, the lines for primary education have been omitted from the figure.

**Fig. 4.**
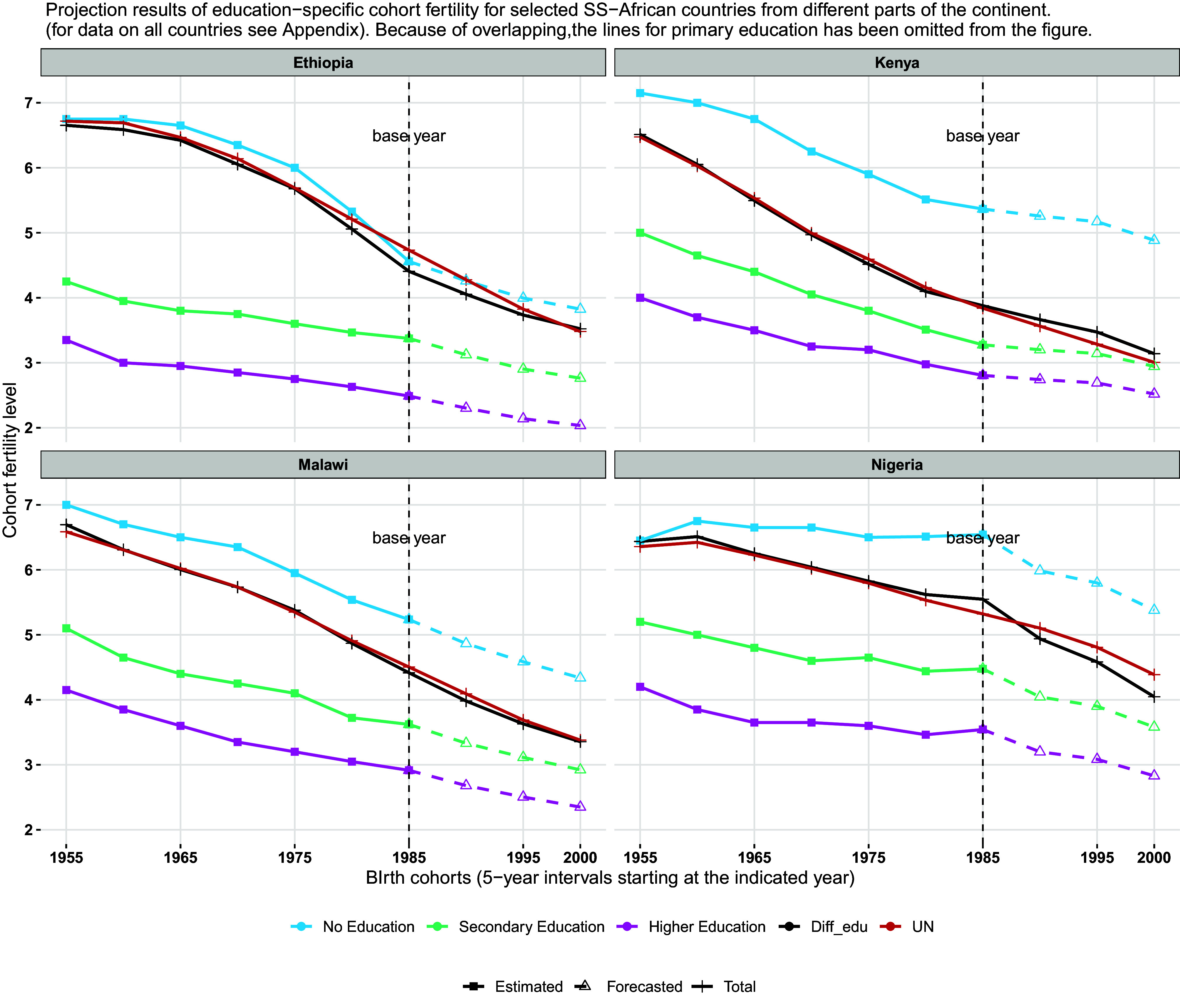
Projection results of education-specific cohort fertility for selected SS-African countries from different parts of the continent.

Fig. 4 shows the projections of completed education-specific cohort fertility trends for four selected countries in SS-Africa: Ethiopia, Kenya, Nigeria, and Malawi. The data for all 37 African countries are given in *SI Appendix*. Ethiopia is the second-largest country of Africa with a population of currently around 120 million forecast to increase to above 200 million. In the birth cohort of 1955–1959, it had one of the world’s highest levels of completed cohort fertility of around seven children per woman which was almost identical to that of uneducated women because there were only tiny elites with some education. But education improved rather rapidly and more-educated women early on had significantly lower fertility. Even the fertility of uneducated cohorts declined significantly from around seven to 4.5 children per woman. In terms of projections, the overall cohort fertility derived from the education-specific projections are virtually identical to those of the UN—which does not explicitly differentiate by education—for the 2000–2005 birth cohort.

Kenya is another interesting case. In the 1970s, it was the country with the highest overall period fertility rate in the world, as given by the World Fertility Survey (39), with a level above eight children per woman and the vast majority of women without formal education. Kenya has also been the subject of important studies highlighting the importance of diffusion in fertility and contraceptive use (30). Another interesting pattern of the education-specific fertility trends is the evident stall of the period fertility decline of uneducated women around the year 2000, which was much more moderate for the more-educated women. This fertility stall in many African countries has widely been discussed in the literature (40–44). But the cohort fertility trends shown in the figure hardly indicate any stall—except for some slow-down in the decline for uneducated women—thus suggesting that the stall was largely a period phenomenon. In terms of the projection of overall cohort fertility, our approach based on education-specific trends results in a slightly higher level than the UN projections.

Malawi is shown as an example of a high-fertility country in the Southern part of the continent. It shows very regular, almost linear declines in education-specific fertility levels. Due to this regularity, the UN projections approach which does not differentiate by level of education results in almost identical forecasts to our method. But this is very different in Africa’s biggest country Nigeria which experienced some discontinuities in education as well as fertility trends. While fertility levels have been unusually high, even at given levels of education, there recently has been a significant expansion of female education—with currently about 45 percent of all women aged 20 to 39 having upper-secondary education—which according to our method will result in a stronger near-term decline than the UN model not considering this education discontinuity. Hence, both the case of Kenya described above and the example of Nigeria show that it does make a difference, when education is explicitly incorporated into fertility projections and thus projections of total population.

While this exercise shows the general feasibility of the proposed projection method, several points still require further analysis and exploration. One question refers to interpreting the trends in ideal and actual fertility in the highest education group. If we were to strictly focus on the diffusion from higher to lower education groups, the highest group should be seen as the avant-garde, whose own trend should hardly be affected by diffusion. However, the empirical analysis clearly shows that fertility in this highest group also declines as a function of increasing mean years of schooling in the cluster. But it is not implausible to assume that highly educated women in a largely uneducated cluster behave differently than in the case where most other women they interact with are rather well educated. There can be a reinforcing effect with feedback from the average behavior. Dasgupta (45) speaks of conformist behavior when the family size that each household desires is positively related to the average. This kind of feedback from the average requires further analysis but is already partly included in the design of this study where we use average education/skills in the cluster/country rather than focusing only on the relative sizes of the different education groups as decomposition studies usually do.

## Materials and Methods

This study combines different sources of data, using both individual-level information from demographic and household surveys as well as national-level time-series data on educational attainment, indicators of quality-adjusted mean years of schooling and education-specific fertility rates.

At the individual level, our analysis pools relevant data on 1.03 million ever-married women aged 15 to 49 from 138 DHSs collected in 39 Sub-Saharan African countries over the years 1986–2022. In each country, the DHS surveys made use of a two-stage cluster sampling design and standard questionnaires to collect comparable nationally representative data on demographic, socioeconomic, and health characteristics of eligible women of reproductive age (15 to 49) (further details are available in ref. 46).

Ideal fertility in DHS data indicates the number of children women (15 to 49) idealized to have in their lifetime regardless of how many children they have had or can have. On the other hand, actual fertility is measured by the total number of live births women aged 40 to 49 (sample = 257,037) say they had over the course of their lives. This analysis focused specifically on ever-married women who provided numerical responses regarding their ideal and actual fertility, allowing us to quantitatively examine fertility ideals and behaviors within the context of marriage. Nonnumerical responses were excluded from our analysis to ensure accurate interpretation during the quantitative analysis.

The study evaluated the importance of diffusion in the fertility transition and operationalized it to population projections using three main steps. First, the diffusion of family size (both ideal and actual) at a strata level was examined by demonstrating how the prevalence of higher-educated women impacts the fertility of lower-educated women residing in the same strata.^*^ This was accomplished by fitting fixed-effect Poisson regression models and estimating the association between individual family size and the mean years of schooling at the strata level. The predictors in the analysis are strata-level mean years of schooling (MYS), individual education (classified as no formal education, primary education, secondary education, and higher education), and the interaction between the two. The model used to predict education-specific ideal and actual family size at different levels of strata level mean years of schooling is

$$\begin{array}{c} log\left(I{\mathrm{FS}}_{i,s}\right)=\beta 0+\beta 1\;{\mathrm{EDUC}}_{i,s}+\beta 2\;{\mathrm{MYS}}_{s}+\beta 3\;{\mathrm{EDUC}}_{i,s}*{\mathrm{MYS}}_{s}+U_{S}+\varepsilon_{i,s}, \end{array}$$

where ${\mathrm{IFS}}_{\mathrm{is}}$ the expected count of the ideal family size for woman i in stratum s, ${\mathrm{EDUC}}_{i,s}$ denotes a categorical variable for the education level of women woman i in stratum s, the random term $U_{S}$ captures the unobserved strata-specific effects, $\varepsilon_{i,s}$ is the error term, β0,β1,β2, and β3 are parameters to be estimated. The key predictor ${\mathrm{MYS}}_{s}$ stands for the mean years of schooling in a stratum s. The interaction term, ${\mathrm{EDUC}}_{i,s}x{\mathrm{MYS}}_{s}$, allows for the examination of how the relationship between individual women’s education level and their ideal family size varies with the educational context of the stratum.

Predictions are then made by calculating $log\left(I{\mathrm{FS}}_{i,s}\right)$ for the relevant combinations of predictors. To predict the ideal family size at different levels of mean years of schooling in the strata, the estimated coefficients $\hat{\beta }0,\hat{\beta }1,\hat{\beta }2$, and $\hat{\beta }3$ are used along with specific education values of ${\mathrm{EDUC}}_{i,s}$ and ${\mathrm{MYS}}_{s}$. The details of the different model specifications and numerical results are given in *SI Appendix*.

At the aggregate national level, to compute the cohort fertility rate for each education group and country from the period fertility rates available, we begin by defining specific birth cohorts of five years age group (1955–1959, 1960–1964, 1965–1969, 1970–1974, and 1975–1979) born in a base year denoted as *t*. We used education-age-specific period fertility rates (EASFRs) for each relevant age group (15 to 19, 20 to 24, …, 40 to 44) from corresponding years (*t* + 15, *t* + 20, …, *t* + 40) using the recently published education and age-specific fertility data (11). For each birth cohort, we sum the EASFRs across the designated age groups for each education, ensuring the inclusion of rates up to the age of 40 to 44, assuming women complete childbearing by age 40. The observed sum is then multiplied by 5 to reflect the five-year span of each age group.

$${\mathrm{CFR}}_{t,e,c}=5(\sum_{i=15}^{44}{\mathrm{ASFR}}_{i,e,c\;t+i)},$$

where, ${\mathrm{ASFR}}_{i,e,c,t+i}$ is the age and education-specific fertility rate for age group *i* and education *e* in country *c*, at time *t* + *i*.

The similar method is used to convert UN’s period fertility into completed cohort fertility as shown in Figs. 3 and 4.

For the cohort 1980–1984 and 1985–1989, we estimated the cohort fertility using the Lee–Carter method which was further developed by Li and Wu (26) (see the details of the calculation in *SI Appendix*).

Next, we estimate the education-specific “diffusion rate” as defined as the percentage change in education-specific cohort fertility rates for a unit change in national-level skill adjusted mean years of schooling (SLAMYS) of women between the cohorts born in 1955–1959 and 1985–1989 for Africa.

$${\mathrm{Diffrate}}_{e(1955-1985)}=\left\lceil \frac{{\mathrm{CFR}}_{e\left(1955\right)-{\mathrm{CFR}}_{e(1985)}}}{{\mathrm{CFR}}_{e(1955)}}\right\rceil .\frac{1}{{\mathrm{SLAMYS}}_{\left(1985\right)-{\mathrm{SLAMYS}}_{\left(1955\right)}}},$$

where ${\mathrm{Diffrate}}_{e(1955-1985)}$ is the marginal effect of overall improvement in SLAMYS women in Africa on the change in CFR of women of education group e between cohorts of 1955 and 1985.

- $\left\lceil \frac{{\mathrm{CFR}}_{e\left(1955\right)-{\mathrm{CFR}}_{e(1985)}}}{{\mathrm{CFR}}_{e(1955)}}\right\rceil$ is the percentage change in CFR for women of education group e between the cohorts of 1955 and 1985
- ${\mathrm{SLAMYS}}_{\left(1985\right)-{\mathrm{SLAMYS}}_{\left(1955\right)}}$ is the overall change in SLAMYS of women for Africa between cohorts of 1955 and 1985

The details of the estimation procedures are provided in *SI Appendix*.

Finally, we apply the estimated education-specific diffusion rates (step 3) to the projected SLAMYS and calculate the national level time-series of education-specific CFR for the subsequent cohorts as follows:

$$\begin{array}{c} {\mathrm{CFR}}_{e(t+5)}={\mathrm{CFR}}_{e\left(t\right)}\\ \;+\left[{\mathrm{CFR}}_{e\left(t\right)}*{\mathrm{Diffrate}}_{e\left(1955-1985\right)}*\left({\mathrm{SLAMYS}}_{\left(t+5\right)}-{\mathrm{SLAMYS}}_{\left(t\right)}\right)\right], \end{array}$$

where *t* is the birth cohort for projected cohorts, 1985, 1990, ⋯, 2000; ${\mathrm{CFR}}_{e\left(t\right)}$ is the education-specific average CFR for Africa and cohort t; ${\mathrm{CFR}}_{e(t+5)}$ is the estimated education-specific CFR for Africa for the subsequent cohort t + 5; ${\mathrm{SLAMYS}}_{\left(t+5\right)}-{\mathrm{SLAMYS}}_{\left(t\right)}$ is the percentage change in education-specific CFR for a unit change in national-level skill adjusted mean years of schooling of women born in 1955 and 1985 (detailed description is given in *SI Appendix*); and ${\mathrm{SLAMYS}}_{\left(t+5\right)}-{\mathrm{SLAMYS}}_{\left(t\right)}$ is the change in estimated SLAMYS of women in Africa for the subsequent cohort [t,t+5].

To forecast the CFR for each country, we applied the same education-specific diffusion rates estimated for the whole Africa and used SLAMYS specific for each country.

## Supplementary Material

Appendix 01 (PDF)

## Data Availability

Previously published data were used for this work (11, 27, 47, 48).

## Footnotes

^*^Strata is a collection of sample clusters (the smallest geographical or administrative unit used as the primary sampling unit in the survey design). To adequately address spatial and social proximity, we based the analysis on 58,708 clusters and 11,873 strata. The average sample size within each cluster was 27, ranging from 1 to 1,539. Because of partly very small cell size, the analysis presented in this document was mostly carried out at the stratum level where the average sample size was 280 women.
