# Supplementary material for: Forecasting Africa’s fertility decline by female education groups
Source: Proc Natl Acad Sci U S A. 2024 Nov 4;121(46):e2320247121. doi: 10.1073/pnas.2320247121 (PMC11572978; doi:10.1073/pnas.2320247121)
Supplement: Supplementary file 1 — Appendix 01 (PDF) [file pnas.2320247121.sapp.pdf]

PNAS

Supporting Information for
Forecasting Africa's fertility decline by female education groups

Saroja Adhikari, Wolfgang Lutz, Endale Kebede

Wolfgang Lutz, Saroja Adhikari
Email: lutz@iiasa.ac.at, adhikari@demogr.mpg.de

This PDF file includes:

Supporting text
Figures S1 to S5
Tables S1 to S3
SI References

Supporting Information Text

This supplementary information on “Forecasting Africa’s fertility decline by diffusion processes among education groups” provides 1) The detailed description of method used in the main paper 2) Sensitivity analysis conducted to confirm the diffusion effect in fertility and 3) Fertility projection (out of sample projection) using diffusion model for education in Africa.

<u>Survey questions asked in the DHS to measure ideal and completed fertility</u>

This study used survey data from the DHS,including country specific geographical units. These units, known as clusters and strata, vary across countries as well as vary across surveys for the same country.
The education variables, highest educational attainment, of women is constructed based on the following two questions:

I. Have you ever attended school?

If the answer is no, the response is categorized as "No education" in the survey data. If yes, the respondent is asked the following question:

II. What is the highest level of school you attended: primary, secondary, or higher?

Based on the answers to questions I and II, a new variable, "educational attainment," is created in the DHS data set and categorized into "no education," "primary," "secondary," and "higher" education.These educational categories are based on country specific educational system.

We calculated the mean years of schooling for women in a cluster and stratum based on another educational variable which is constructed using the following questions in the DHS data:

III. What is the highest level of school you attended: primary, secondary, or higher?
IV. What is the highest (grade/form/year) you completed at that level?

Based on the responses to questions III and IV, the total number of years spent in school is calculated in DHS datasets. Then we calculated mean years of schooling by averaging these total years spent in school.

The completed fertility in our study is the total number of live children ever born to women of age 40 to 49. This information in DHS is collected by asking women the four questions as follows:

V. How many sons live with you? And how many daughters live with you?
VI. How many sons are alive but do not live with you? And how many daughters are alive but do not live with you?
VII. Have you ever given birth to a boy or girl who was born alive but later died?
VIII. If yes to question number VII. How many boys have died? And how many girls have died? If no to question number III, put zero

Using these four question DHS calculated the new variables of total number of children ever born to women by adding the number provided by women in questions V, VI and VII.

The ideal fertility used from the DHS survey is the number of children women idealized to have regardless of how many they have or can have. Information about the ideal number of children is collected by asking women who already have children the following questions:

IX. If you could go back to the time, you did not have any children and could choose exactly the number of children to have in your whole life, how many would that be?

For those who do not have living children, the question is phrased a little differently:

X. If you could choose exactly the number of children to have in your whole life, how many would that be?

Data and Method

The microdata used in this study were obtained from 138 Demographic and Health surveys conducted in 39 sub-Sahara African countries between 1986 and 2022 with a range of about 5 years. SI appendix table S.1 reports the sample countries and respective survey rounds included in the dataset. The DHS use a two-stage cluster sampling procedure to collect nationally representative indicators of demographic, health and living conditions of individuals and households (1). In the first stage, sample frames were constructed by stratifying the population by key demographics, using the most recent census data. This stratification divides the country into primary sampling units, known as clusters, which are subsequently selected based on a probability proportional to their population size. The subsequent stage involves conducting a comprehensive household listing within each selected cluster, from which approximately 20 to 30 households are chosen for in-depth, face-to-face interviews with eligible members. The total sample sizes can range from 4,000 to 30,000 households depending on the country's population size. Our pooled dataset includes 1.03 million ever-married women aged 15-49, distributed across 11,873 strata and 58773 clusters, with each stratum consisting of several smaller clusters (~4 or ~5 clusters).

Our outcome variable, the ideal family size, measure the number of children a woman idealized to have regardless of how many they have or can have. To examine the effect of women education on own fertility desires, we categorize it into four levels as reported in the DHSs: no education, primary education, secondary education, and higher education (above secondary education). We have also considered the independent and diffusion effect of education of others, and therefore, we have calculated the mean years of completed schooling (MYS) of women in each sample stratum.

Statistical Methods

We estimate a fixed effect Poisson regression model to predict the ideal family size of woman i with in a stratum s (IFS_{is}). This approach was selected due to the count nature of the outcome variable (ideal family size) and the need to control for unobserved stratum level determinants. The Model incorporates individual level education and strata level mean years of schooling, along with their interaction to predict IFS_{is}. The model can then be specified as:

$$log\left(IFS_{i,s}\right) = \beta 0 + \beta 1\ EDUC_{i,s} + \beta 2\ MYS_s + \beta 3\ EDUC_{i,s} *\ MYS_s + U_S + \varepsilon_{i,s}$$ --------------(eq.S.1)

Where IFS_{is} the expected count of the ideal family size for woman i in stratum s, $EDUC_{i,s}$ denotes a categorical variable for the education level of women woman i in stratum s, the random term U_S captures the unobserved strata-specific effects, $\varepsilon_{i,s}$ is the error term, $\beta 0, \beta 1$, $\beta 2$, and $\beta 3$ are parameters to be estimated. The key predictor MYS_s stands for the mean years of schooling of women age 15-49 years in a stratum s. The interaction term, $EDUC_{i,s}x\ MYS_s$, allows for the examination of how the relationship between individual women's education level and their ideal family size varies with the educational context of the stratum.

Predictions are then made by calculating $log\left(IFS_{i,s}\right)$ for the relevant combinations of predictors. To predict the ideal family size at different levels of mean years of schooling in the strata, the estimated coefficients $\hat{\beta} 0, \hat{\beta} 1$, $\hat{\beta} 2$, and $\hat{\beta} 3$ are used along with specific education values of $EDUC_{i,s}$ and MYS_s.

We used the same model to predict the completed cohot fertility, we replaced the dependent variable IFS_{is} in eq. S.1 by total number of live chidren ever born per women aged 40-49 years and the MYS_s is also calculated for women aged 40-49 years living in the stratum s.

Appendix Table S.1 presents the results of a fixed effect Poisson model (eq.S.1) regression analysis that examines the relationship between individual and community level education and women's ideal family size (aged 15-49) as well as the number of children ever born (aged 40-44). The estimates of the analysis are presented as the incidence rate ratio (IRR) along with 95% confidence intervals. The results indicate that the ideal family size of women who have not received any formal education is 0.78 and 0.7 times higher than those with a secondary and higher level of education, respectively. Similarly, women with a secondary and higher level of education tend to have 0.84 and 0.62 times fewer children over their lifetime than their uneducated counterparts. Furthermore, within the same group of women, completed fertility decreases by 0.98 and 0.99 times for each unit change in the mean years of schooling (MYS) of the strata in which they reside.

Table S1. Incident rate ratio for ideal and actual family size from model of eq S.1

Variables	Labels	IRR (LL-UL)	
		Ideal family size	Completed family size
Education (ref="No education")	Constant	6.684***(6.64-6.729)	6.858***(6.821-6.896)
	Primary Education	0.899***(0.895-0.902)	0.995(0.988-1.003)
	Secondary Education	0.785***(0.78-0.79)	0.845***(0.833-0.856)
	Higher Education	0.699***(0.686-0.712)	0.626***(0.605-0.648)
Community education	MYS community	0.96***(0.958-0.961)	0.977***(0.975-0.979)
Interaction effect	Primary: MYS community	0.999***(0.997-1)	0.991***(0.989-0.993)
	Secondary: MYS community	0.998**(0.997-1)	0.987***(0.984-0.989)
	Higher: MYS community	1.002(0.999-1.005)	0.995**(0.99-1)

*** p<0.001; ** p<0.05; * p< 0.1

Estimating Cohort Incomplete Fertility

To compute the cohort fertility rate for each education group and country from the period total fertility rate, we begin by defining specific birth cohorts of five years age group (1955-59,1960-64,1965-69,1970-74 and 1975-79) , born in a specific year denoted as t. We used education-age-specific period fertility rates (EASFRs) for each relevant age group (15-19, 20-24, ..., 40-44) from corresponding years (t+15, t+20, ..., t+40) using the recently published education and age specific fertility data (2). For each birth cohort, we sum the EASFRs across the designated age groups for each education, ensuring the inclusion of rates up to the age of 40-44, assuming women complete childbearing by age 40. The observed sum is then multiplied by 5 to reflect the five-year span of each age group.

$$CFR_{t,e,c} = 5(\sum_{i=15}^{44} ASFR_{i,e,c\ t+i\ )} \text{------------------}(1)$$

Where, t=1955-1959, 1960-1964,---1975-1979.

$ASFR_{i,e,c,t+i}$ is the age and education specific fertility rate for age group i and education e in country c at time t+i.

We estimated incomplete fertility rates for the 1980-84 and 1985-89 birth cohorts using the Lee-Carter method (3) which was further developed by (4). To do this, we specified the Age-Specific Fertility Rate (ASFR) of a cohort and five-year age group as:

$$f(x,t) = a(x) + b_x k_t + \varepsilon_{x,t} --------(2)$$

Here, $a(x)$ represents the mean age-specific fertility rate over completed cohorts, b_x is the average contribution of age group x to overall fertility changes over the completed birth cohorts (1955/59-1975/79), and k_t represent the incremental change in the Total Fertility Rate (CTFR) in cohort t . While $\hat{a}(x)$ were easily obtained by averaging the cohort age-specific fertility rates of the completed cohorts, $\hat{b}_x$ and $\hat{k}_t$ were estimated via a Singular Value Decomposition (SVD) of the ASFR matrix for the completed observations.

Using the SVD method proposd by (3), we decomposed the cohort ASFR matrix into:

$$M = UDV^T$$

Where $D -$ is a diagonal matrix containing singular values, and U and V are orthogonal matrices known as the left and right singular vectors, respectively. $\hat{b}_x$ was then estimated by taking the first column of the first right singular vector (V), which was normalized such that its elements summed up to one.

Once $\hat{a}(x)$ and $\hat{b}_x$ were estimated, $\hat{k}_t$ could be estimated by fitting equation 1 using Ordinary Least Square (OLS) method as:

$$\hat{k}_t = \frac{\sum_{x=15}^{44}[f(x,t) - \hat{a}(x)]\hat{b}_x}{\sum_{x=15}^{44} \hat{b}_x{}^2} ------------(3)$$

The next step was to estimate incomplete fertility rates of the younger cohorts by applying the established relationship between the level and age pattern of fertility for completed cohorts. For the 1980/85 birth cohort with complete information of ASFR for age group 15-39:

$$f(40{,}1980) = \hat{a}(x) + \hat{b}_x \hat{k}_{1980} --------(4)$$

$$\hat{k}_{1980} = \frac{\sum_{x=15}^{39}[f(x,1980) - \hat{a}(x)]\hat{b}_x}{\sum_{x=15}^{39} \hat{b}_x{}^2} ------------(4.1)$$

Similar strategy was also employed to estimate $f(40{,}1985)$ and $f(35{,}1985)$ for each sample country and education group.

Estimating the education specific 'diffusion rate'

We have estimated the education-specific average "diffusion rate" for Africa. This refers to the percentage change in the education-specific average (over sample countries) CFR (childbearing total fertility rate) for a unit change in the average cohort-specific Skill in Literacy Adjusted Mean Years of Schooling (SLAMYS) of women between the cohorts born in 1955 and 1985.

However, the SLAMYS data available (5) only provides period information. Therefore, we had to obtain the cohort SLAMYS through the following steps: First, we calculated a skill adjustment factor for each period and age group. This was done by calculating the ratio of SLAMYS of each country in a given year to the national level MYS (mean years of schooling) for the same age group in the same year, taken from the Wittgenstein Centre Human Capital Data Explorer. Second, we converted the period skill adjustment factors to cohort-specific skill adjustment factors. This was done by conducting a retrospective assignment. We attributed the skill adjustment factor estimated for a given year and age group to the cohort born 15 years prior. For example, the skill adjustment factor estimated for the year 1970 and age 15-64 was attributed to the cohort born in 1955. Third, we calculated the national level cohort SLAMYS for women by multiplying the cohort skill adjustment factors by the MYS of the same cohort of women when they were between the ages of 25 and 29 years.

In an effort to translate the examined diffusion effects into the national level fertility forecasting, we computed the marginal effect of changes in national-level SLAMYS for women between the cohort born in 1955 and 1985 to the change in cohort fertility rate (CFR) of same birth cohorts. We chose this observation window based on the availability of consistent time series data on education-specific cohort fertility rates, which began in 1955. We estimated the education-specific "diffusion rates" as the percentage change in average CFR in Africa for a unit change in SLAMYS of women in Africa as follow:

$$Diffrate_{e(1955-1985)} = \left[\frac{CFR_{e(1955)} - CFR_{e(1985)}}{CFR_{e(1955)}}\right] \cdot \frac{1}{SLAMYS_{(1985)} - SLAMYS_{(1955)}} \text{------------------(eq.S.2)}$$

Where : - $Diffrate_{e(1955-1985)}$ is the marginal effect of overall improvement in SLAMYS women in Africa on the change in CFR of women of eduation group e between cohorts of 1955 and 1985.

- $\left[\frac{CFR_{e(1955)} - CFR_{e(1985)}}{CFR_{e(1955)}}\right]$ is the percentage change in CFR for women of education group e between the cohorts of 1955 and 1985
- $SLAMYS_{(1985)} - SLAMYS_{(1955)}$ is the overall change in SLAMYS of women for Africa between cohorts of 1955 and 1985

1. **Incorporating the diffusion process in the national level fertility forecasting**

To incorporate the diffusion in the national-level fertility forecasting model, we apply the above estimated “education specific diffusion rates” to the projected SLAMYS for the female cohort born in 1985 to 2000 as follows:

$$CFR_{e(t+5)} = CFR_{e\,(t)} + \left[CFR_{e,(t)} * Diffrate_{\,e\,(1955-1985)} * \left(SLAMYS_{\,(t+5)} - SLAMYS_{\,(t)}\right)\right] \text{------------ (eq.S.3)}$$

Where: $t\ is\ \ the\ birth\ cohort\ for\ projected\ cohorts\ , 1985, 1990, \ldots \ldots . .2000$

: $CFR_{e(t)}$ $is\ the$ education specific average CFR for Africa and cohort t .

: $CFR_{e(t+5)}$ is the estiamted education specific CFR for Africa for the subsequent cohort t+5.

: $Diffrate\ _{e(1955-1985)}$ is the percentage change in education specific CFR for a unit change in national level skill adjusted mean years of schoolingof women born in 1955 and 1985, as described in (eq.S.2)

: $SLAMYS_{(t+5)} - SLAMYS_{(t)}$ is the change in estimated SLAMYS of women in Africa for the subsequent cohort $[t, t+5]$

Overall CFR (without education) for each projected cohorth from 1985 to 2000 was computed using a similar strategy, but the diffusion rates averaged across education groups and countries and is named as “Diff_tot”.

Sensitivity analysis

To test the diffusion effect in fertility intention and behavior we performed three different sensitivity analysis. In figure S1(a) and Figure S1 (b) we test the effect of individual education and stratum MYS on ideal and completed fertility using model S.1 but we added rural and urban place of residence as additional predictors. These aimed to explore whether this decline was a result of diffusion or influenced by other factors, such as urbanization and socio-economic developments over time. Our analyses consistently revealed similar patterns with and without accounting for area of residence. Figure S1 and Figure S1 (b) illustrate the ideal and actual fertility among women with all four levels of education, separately for rural and urban regions. This pattern closely resembles the one presented in Figure 1 of the main text, with a slightly higher actual fertility among less educated women in rural areas and slightly lower fertility levels in urban areas as expected. The figures also support our hypothesis that the pattern emerges from the diffusion effect.

We conducted further tests on the results presented in Figure 1 of the main text to investigate whether the observed pattern, attributed to diffusion, could be affected by changing socio-economic factors like increased access to contraception or shifts in the economy over time. To do this, we narrowed our analysis to only include the most recent Demographic and Health Surveys (DHS) data for each country, collected after 2010. We then used this data to illustrate fertility trends based on women's education and mean years of schooling (MYS) within specific strata. Figure S2 shows the results of this analysis. Once again, we observed a consistent gradient and pattern in fertility, affirming that the declining fertility associated with women's education and the additional decline linked to increasing MYS within specific strata are indeed the result of the diffusion effect.

Figure S3 displays a diffusion pattern closely resembling the one illustrated in Figure 1 of the main paper. However, it is crucial to note a distinction: the diffusion pattern in Figure S3 is observed at the cluster level, the smallest geographic unit in the DHS, as opposed to the stratum level in Figure 1 of the main paper. Despite this difference in geographic units, the overall trends remain remarkably similar. Both patterns reveal a gradual decline in both ideal and actual fertility with increasing mean years of schooling (MYS) at the community level (stratum or cluster). Notably, at the cluster level, the decline in fertility is more pronounced, indicating rapid diffusion of smaller family concepts and behavior within these smaller geographic clusters.

Figure S4 displays the observed (until 1985/89 birth cohort) and projected education specific cohort fertility (CFR) trends. The UN estimates and projections of overall CFR are represented by the red line. The purple line (Diff_tot) shows the projections derived by applying the estimated

average diffusion rate to the empirical overall CFR of 1985/89 cohort, without differentiating by education level. The black line (Diff-ed) represents the projections derived by applying the average diffusion effect to education-specific CFRs for four education levels and then aggregating them to overall CFRs. This line also accounts for the changing proportions of different education groups (composition effect) according to the SSP2 (medium) education expansion scenario. The education-specific diffusion rates used are 10.02%, 11.09%, 11.57% and 11.64% for no education, primary education, secondary education and higher education, respectively. The primary education line has been omitted from the figure for better visualization.

To test the good fit of our proposed method, we did a so-called out of sample projection by case taking the empirical time-series of education-specific cohort fertility for only a sub-period and apply the method to project the series for the rest of the period for which empirical data are available. Figure S5 are isomorphic to those in Figure 3 from the main text but with shifting the base year to 1975 instead of 1965 and forecasting the CFR for 1980 and 1985 based on the empirical time series from 1955 to 1975.
Solid lines for each education represent estimated fertility rates, while dotted lines show forecasted result. Forecasted CFRs closely matched the estimated fertility rates, confirming again the good fit of the proposed forecasting method.

Fig. S1 (a). DHS, ideal Family size (left) and completed fertility (right) by education groups andMYS in Rural Stratum by including data collected between 1986 and 2022

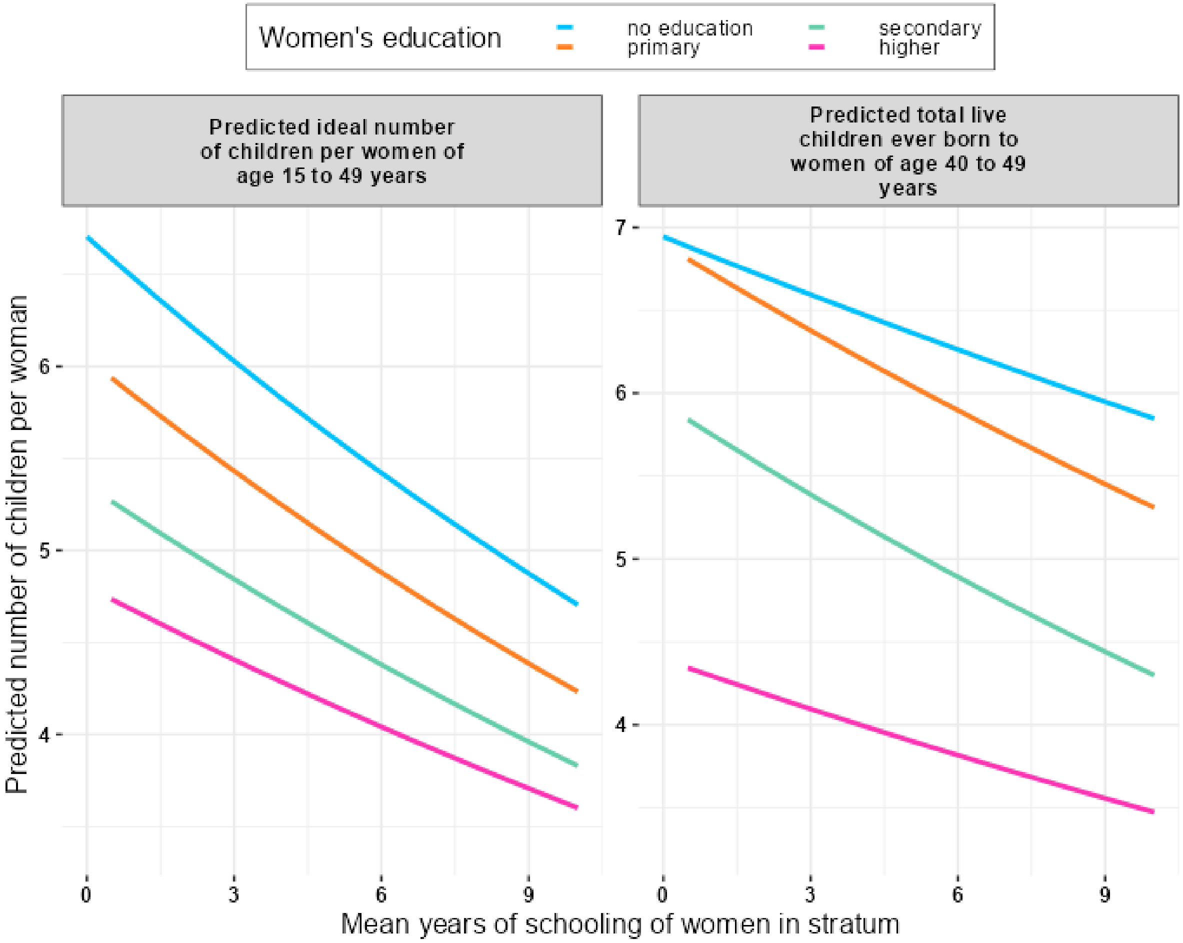

Fig. S1 (b). DHS, ideal Family size (left) and completed fertility (right) by education groups and MYS in Urban Stratum by including data collected between 1986 and 2022

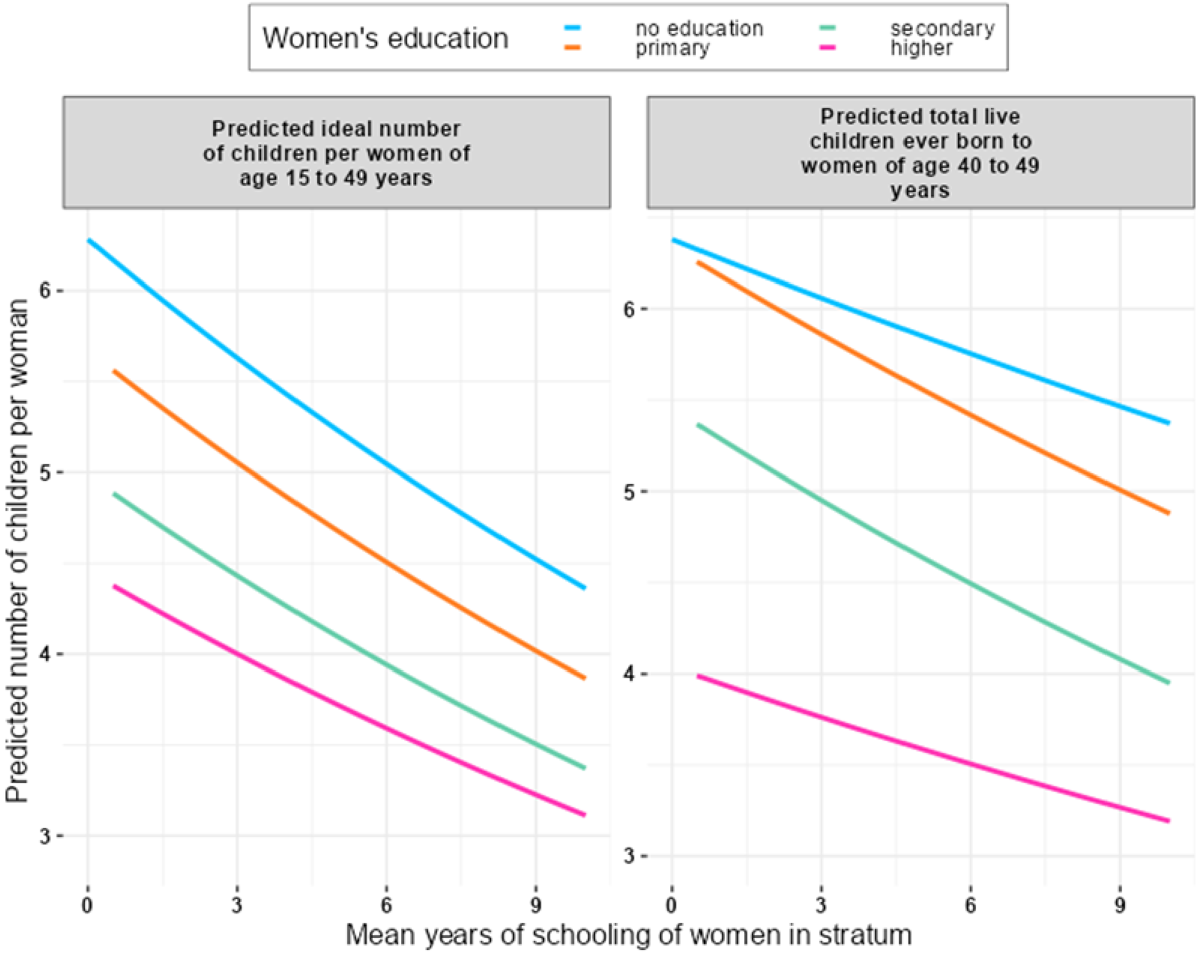

Fig. S2. DHS, ideal Family size (left) and completed fertility (right) by education groups and MYS in Stratum, used only latest survey data from 2010 to 2022.

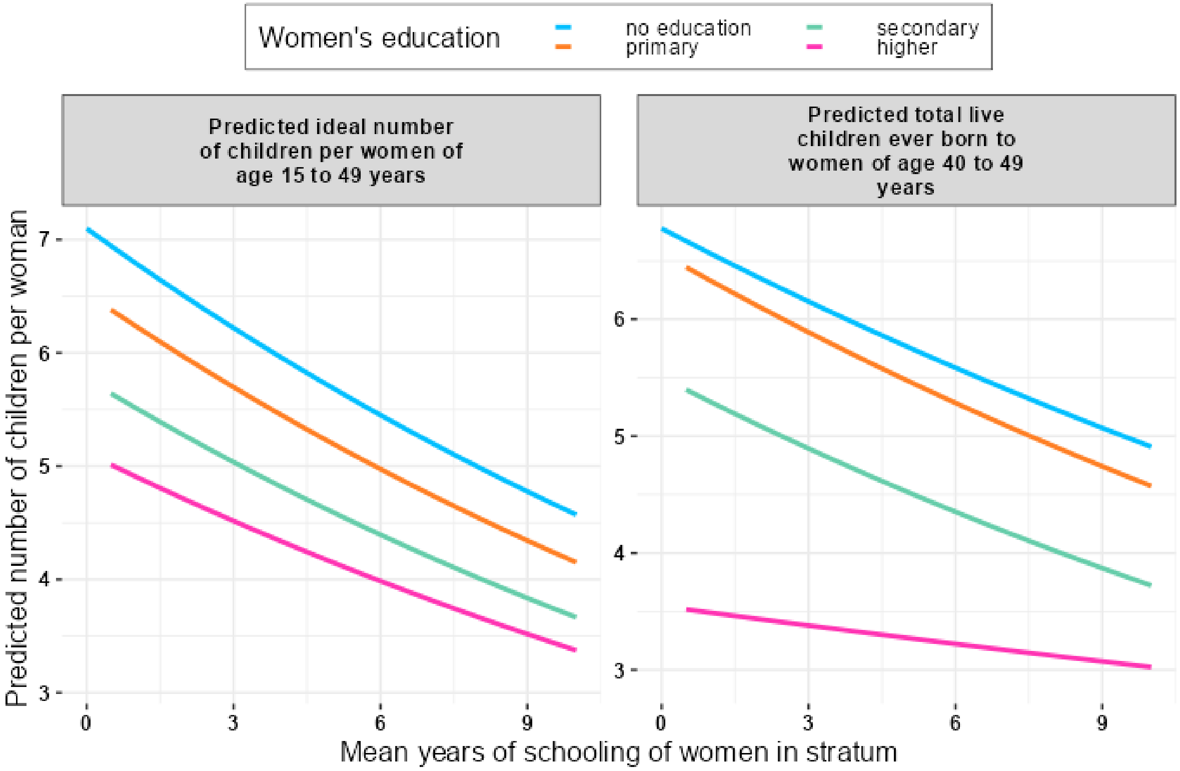

Fig. S3. DHS, ideal Family size (left) and completed fertility (right) by education groups and MYS in Cluster, used survey data from 1986 to 2022.

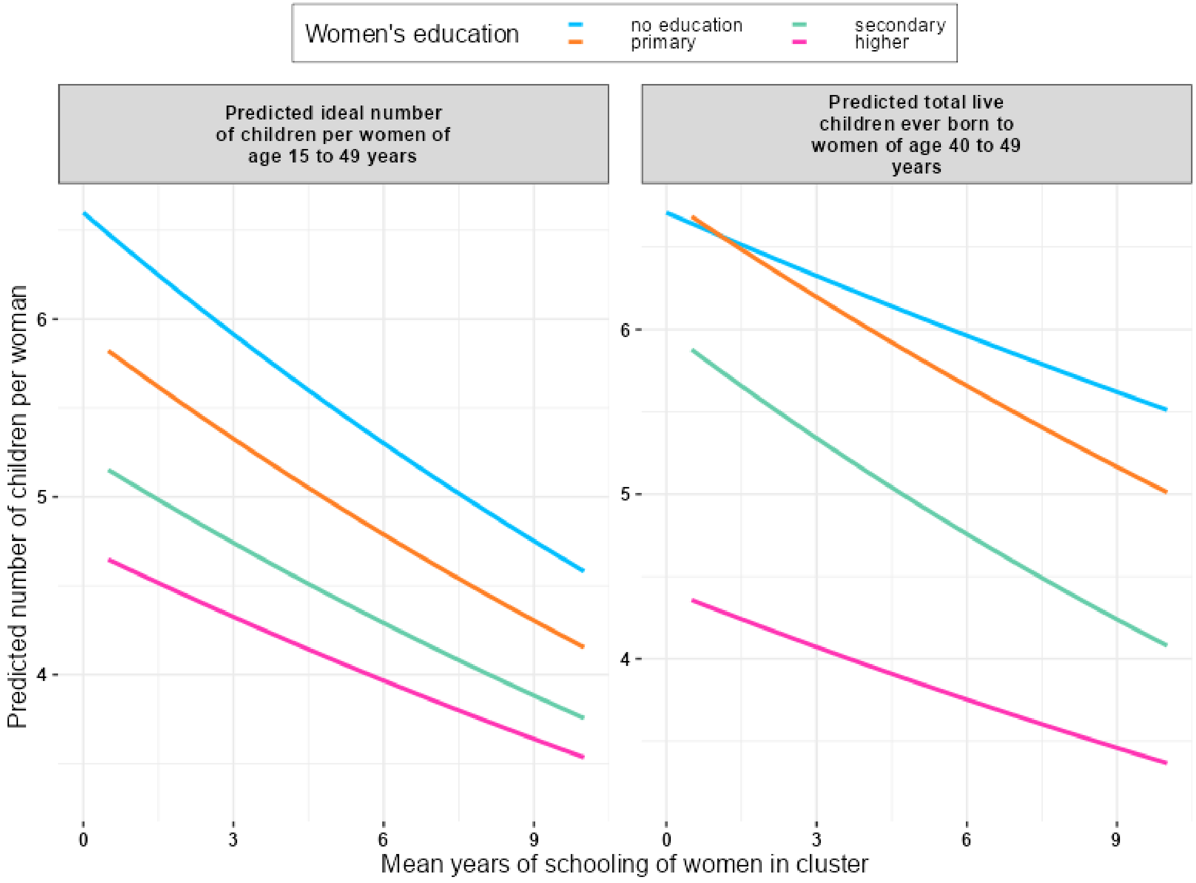

Fig. S4. Cohort fertility rate forecasts for each education in Africa based on diffusion effect and average SLAMYS of Africa.

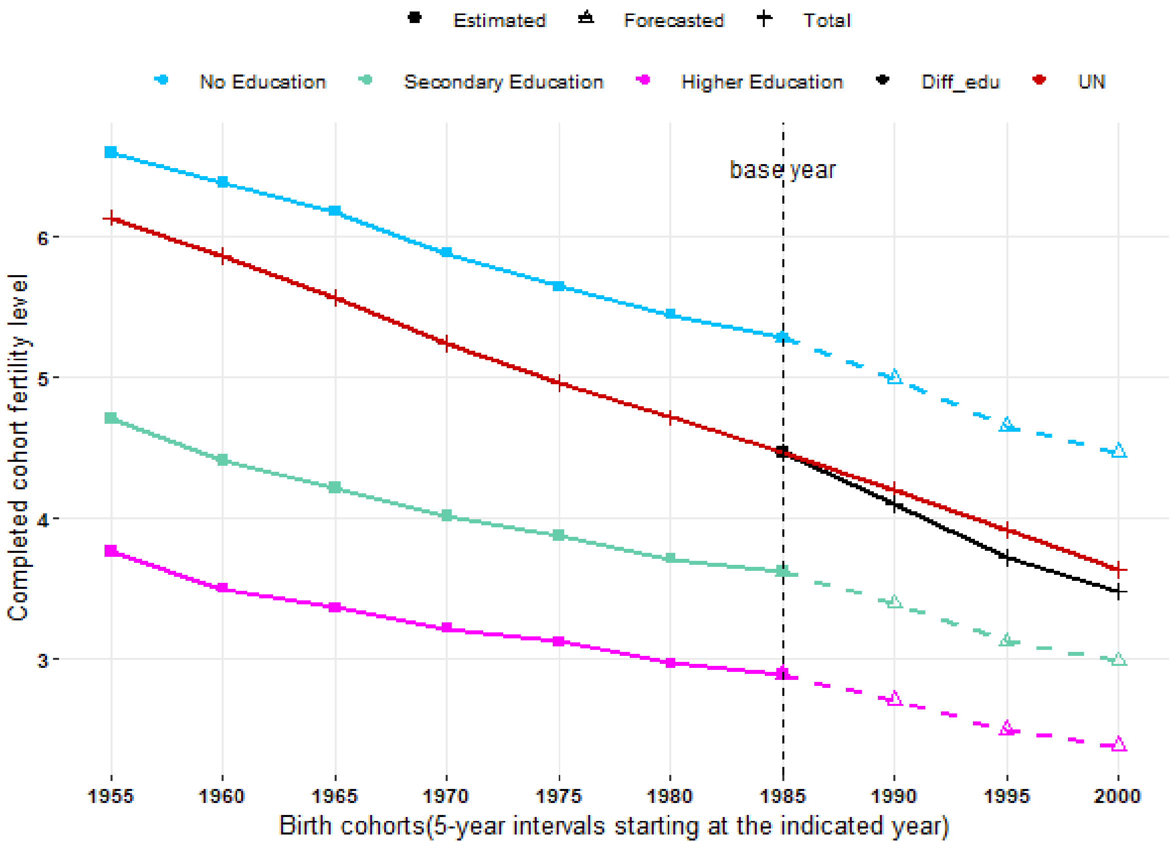

Fig. S5. Cohort fertility rate out of sample forecasts from 1975 to 1985 for Africa

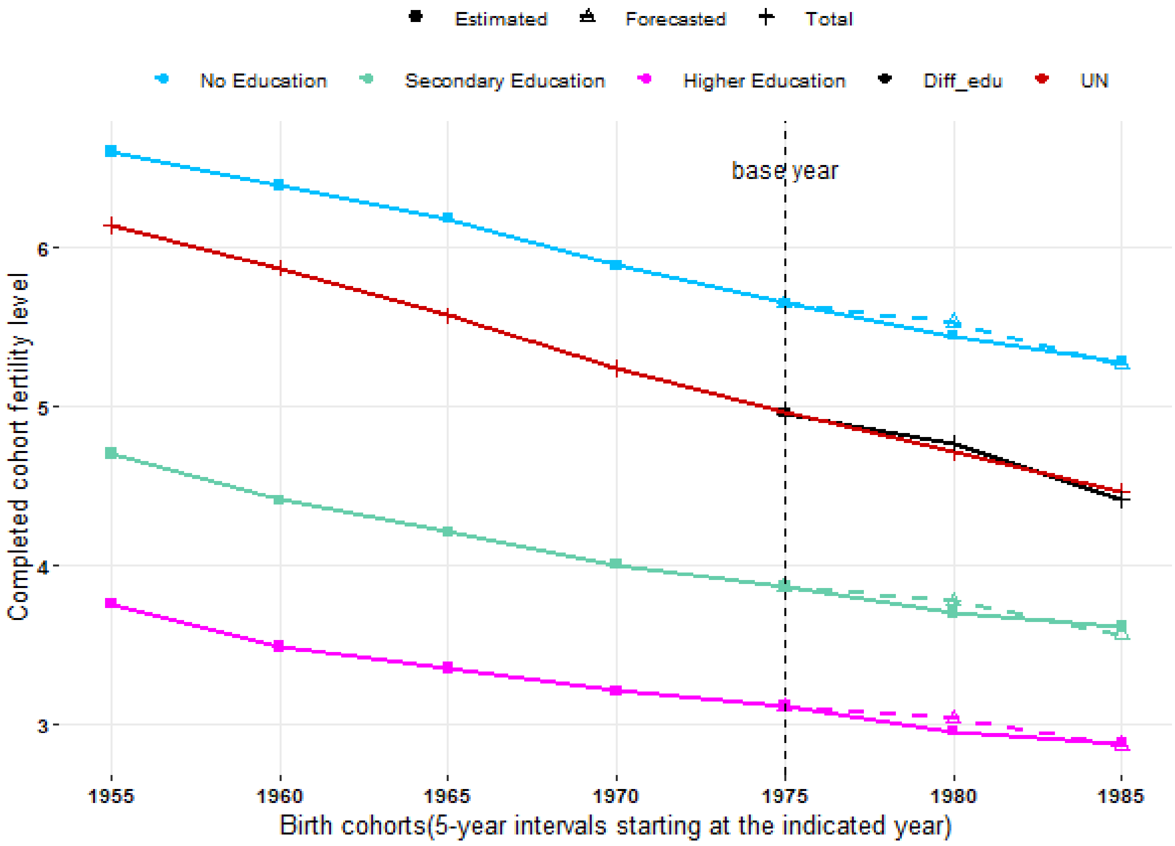

Table S2. List of country and survey year from DHS included in this study.

Country	Survey Years						
Angola	2015						
Benin	1996	2001	2006	2012	2017		
Burkina Faso	1993	1999	2003	2010			
Burundi	1987	2010	2016				
Cameroon	1991	1998	2004	2011	2018		
Central African Republic	1994						
Chad	1997	2004	2014				
Comoros	1996	2012					
Congo	2005	2011					
Congo Democratic Republic	2007	2013					
Cote d'Ivoire	1994	1998	2012				
Eswatini	2006						
Ethiopia	2000	2005	2011	2016	2019		
Gabon	2000	2012	2019				
Gambia	2013	2019					
Ghana	1988	1993	1998	2003	2008	2014	
Guinea	1999	2005	2012	2018			
Kenya	1989	1993	1998	2003	2008	2014	2022
Lesotho	2004	2009	2014				
Liberia	1986	2007	2013	2019			
Madagascar	1992	1997	2004	2008	2021		
Malawi	1992	2000	2004	2010	2015		
Mali	1987	1996	2001	2006	2012	2018	
Mauritania	2020						
Mozambique	1997	2003	2011				
Namibia	1992	2000	2006	2013			
Niger	1992	1998	2006	2012			
Nigeria	1990	2003	2008	2013	2018		
Rwanda	1992	2000	2005	2008	2010	2015	2019
Sao Tome and Principe	2008						
Senegal	1986	1993	1997	2005	2010	2012	2014-2019
Sierra Leone	2008	2013	2019				
South Africa	1998	2016					
Sudan	1990						
Tanzania	1992	1996	1999	2004	2010	2015	
Togo	1988	1998	2013				
Uganda	1988	1995	2000	2006	2011	2016	
Zambia	1992	1996	2002	2007	2013	2018	
Zimbabwe	1988	1994	1999	2005	2010	2015	

Table S3. Estimated and Projected Cohort TFR by education

Country	Education	Cohort	TFR
Angola	No Education	1955	7.000
Angola	No Education	1960	6.800
Angola	No Education	1965	6.700
Angola	No Education	1970	6.700
Angola	No Education	1975	6.600
Angola	No Education	1980	6.416
Angola	No Education	1985	6.084
Angola	Secondary Education	1955	5.250
Angola	Secondary Education	1960	4.750
Angola	Secondary Education	1965	4.650
Angola	Secondary Education	1970	4.500
Angola	Secondary Education	1975	4.450
Angola	Secondary Education	1980	4.382
Angola	Secondary Education	1985	4.366
Angola	Higher Education	1955	4.050
Angola	Higher Education	1960	3.750
Angola	Higher Education	1965	3.750
Angola	Higher Education	1970	3.650
Angola	Higher Education	1975	3.700
Angola	Higher Education	1980	3.547
Angola	Higher Education	1985	3.607
Benin	No Education	1955	6.850
Benin	No Education	1960	6.550
Benin	No Education	1965	6.200
Benin	No Education	1970	5.800
Benin	No Education	1975	5.700
Benin	No Education	1980	5.692
Benin	No Education	1985	5.614
Benin	Secondary Education	1955	4.600
Benin	Secondary Education	1960	4.200
Benin	Secondary Education	1965	4.000
Benin	Secondary Education	1970	3.750
Benin	Secondary Education	1975	3.750
Benin	Secondary Education	1980	3.605
Benin	Secondary Education	1985	3.605
Benin	Higher Education	1955	3.550
Benin	Higher Education	1960	3.400
Benin	Higher Education	1965	3.200
Benin	Higher Education	1970	3.100

Country	Education	Cohort	TFR
Benin	Higher Education	1975	3.100
Benin	Higher Education	1980	2.927
Benin	Higher Education	1985	2.844
Burkina Faso	No Education	1955	6.950
Burkina Faso	No Education	1960	6.700
Burkina Faso	No Education	1965	6.550
Burkina Faso	No Education	1970	6.300
Burkina Faso	No Education	1975	6.050
Burkina Faso	No Education	1980	5.762
Burkina Faso	No Education	1985	5.508
Burkina Faso	Secondary Education	1955	4.950
Burkina Faso	Secondary Education	1960	4.400
Burkina Faso	Secondary Education	1965	4.200
Burkina Faso	Secondary Education	1970	4.050
Burkina Faso	Secondary Education	1975	3.900
Burkina Faso	Secondary Education	1980	3.725
Burkina Faso	Secondary Education	1985	3.655
Burkina Faso	Higher Education	1955	3.950
Burkina Faso	Higher Education	1960	3.600
Burkina Faso	Higher Education	1965	3.450
Burkina Faso	Higher Education	1970	3.100
Burkina Faso	Higher Education	1975	3.100
Burkina Faso	Higher Education	1980	2.958
Burkina Faso	Higher Education	1985	2.971
Burundi	No Education	1955	7.150
Burundi	No Education	1960	7.200
Burundi	No Education	1965	6.950
Burundi	No Education	1970	6.700
Burundi	No Education	1975	6.400
Burundi	No Education	1980	6.284
Burundi	No Education	1985	5.729
Burundi	Secondary Education	1955	5.250
Burundi	Secondary Education	1960	4.800
Burundi	Secondary Education	1965	4.800
Burundi	Secondary Education	1970	4.650
Burundi	Secondary Education	1975	4.600
Burundi	Secondary Education	1980	4.411
Burundi	Secondary Education	1985	4.291
Burundi	Higher Education	1955	4.200
Burundi	Higher Education	1960	4.050

Country	Education	Cohort	TFR
Burundi	Higher Education	1965	3.950
Burundi	Higher Education	1970	3.850
Burundi	Higher Education	1975	3.750
Burundi	Higher Education	1980	3.386
Cameroon	No Education	1955	6.950
Cameroon	No Education	1960	6.700
Cameroon	No Education	1965	6.500
Cameroon	No Education	1970	6.350
Cameroon	No Education	1975	6.050
Cameroon	No Education	1980	6.008
Cameroon	No Education	1985	5.739
Cameroon	Secondary Education	1955	4.550
Cameroon	Secondary Education	1960	4.450
Cameroon	Secondary Education	1965	4.350
Cameroon	Secondary Education	1970	4.250
Cameroon	Secondary Education	1975	4.200
Cameroon	Secondary Education	1980	4.292
Cameroon	Secondary Education	1985	4.169
Cameroon	Higher Education	1955	3.700
Cameroon	Higher Education	1960	3.500
Cameroon	Higher Education	1965	3.450
Cameroon	Higher Education	1970	3.350
Cameroon	Higher Education	1975	3.250
Cameroon	Higher Education	1980	3.226
Cameroon	Higher Education	1985	3.235
Central African Republic	No Education	1955	6.000
Central African Republic	No Education	1960	6.000
Central African Republic	No Education	1965	6.150
Central African Republic	No Education	1970	6.300
Central African Republic	No Education	1975	6.200
Central African Republic	No Education	1980	5.941
Central African Republic	No Education	1985	5.793
Central African Republic	Secondary Education	1955	4.500
Central African Republic	Secondary Education	1960	4.500
Central African Republic	Secondary Education	1965	4.500
Central African Republic	Secondary Education	1970	4.500
Central African Republic	Secondary Education	1975	4.600
Central African Republic	Secondary Education	1980	4.497
Central African Republic	Secondary Education	1985	4.069
Central African Republic	Higher Education	1955	3.550

Country	Education	Cohort	TFR
Central African Republic	Higher Education	1960	3.450
Central African Republic	Higher Education	1965	3.400
Central African Republic	Higher Education	1970	3.500
Central African Republic	Higher Education	1975	3.450
Central African Republic	Higher Education	1980	3.213
Central African Republic	Higher Education	1985	2.928
Chad	No Education	1955	6.800
Chad	No Education	1960	7.150
Chad	No Education	1965	7.500
Chad	No Education	1970	7.500
Chad	No Education	1975	7.100
Chad	No Education	1980	6.928
Chad	No Education	1985	6.941
Chad	Secondary Education	1955	5.200
Chad	Secondary Education	1960	5.100
Chad	Secondary Education	1965	5.150
Chad	Secondary Education	1970	5.200
Chad	Secondary Education	1975	5.250
Chad	Secondary Education	1980	5.048
Chad	Secondary Education	1985	4.926
Chad	Higher Education	1955	4.050
Chad	Higher Education	1960	3.900
Chad	Higher Education	1965	3.900
Chad	Higher Education	1970	4.050
Chad	Higher Education	1975	4.100
Chad	Higher Education	1980	3.830
Chad	Higher Education	1985	3.580
Comoros	No Education	1955	6.850
Comoros	No Education	1960	6.600
Comoros	No Education	1965	6.050
Comoros	No Education	1970	5.600
Comoros	No Education	1975	5.400
Comoros	No Education	1980	5.431
Comoros	No Education	1985	5.487
Comoros	Secondary Education	1955	4.350
Comoros	Secondary Education	1960	4.150
Comoros	Secondary Education	1965	4.000
Comoros	Secondary Education	1970	3.750
Comoros	Secondary Education	1975	3.650
Comoros	Secondary Education	1980	3.546

Country	Education	Cohort	TFR
Comoros	Secondary Education	1985	4.066
Comoros	Higher Education	1955	3.550
Comoros	Higher Education	1960	3.300
Comoros	Higher Education	1965	3.150
Comoros	Higher Education	1970	3.050
Comoros	Higher Education	1975	2.950
Comoros	Higher Education	1980	2.935
Comoros	Higher Education	1985	3.369
Congo	No Education	1955	6.300
Congo	No Education	1960	6.200
Congo	No Education	1965	6.150
Congo	No Education	1970	5.850
Congo	No Education	1975	5.700
Congo	No Education	1980	5.590
Congo	No Education	1985	5.663
Congo	Secondary Education	1955	4.450
Congo	Secondary Education	1960	4.200
Congo	Secondary Education	1965	4.000
Congo	Secondary Education	1970	3.950
Congo	Secondary Education	1975	3.750
Congo	Secondary Education	1980	3.750
Congo	Secondary Education	1985	3.755
Congo	Higher Education	1955	3.550
Congo	Higher Education	1960	3.300
Congo	Higher Education	1965	3.350
Congo	Higher Education	1970	3.150
Congo	Higher Education	1975	3.100
Congo	Higher Education	1980	2.920
Congo	Higher Education	1985	2.835
Côte d’Ivoire	No Education	1955	7.050
Côte d’Ivoire	No Education	1960	6.800
Côte d’Ivoire	No Education	1965	6.450
Côte d’Ivoire	No Education	1970	6.200
Côte d’Ivoire	No Education	1975	5.750
Côte d’Ivoire	No Education	1980	5.407
Côte d’Ivoire	No Education	1985	5.461
Côte d’Ivoire	Secondary Education	1955	4.900
Côte d’Ivoire	Secondary Education	1960	4.500
Côte d’Ivoire	Secondary Education	1965	4.100
Côte d’Ivoire	Secondary Education	1970	3.950

Country	Education	Cohort	TFR
Côte d'Ivoire	Secondary Education	1975	3.800
Côte d'Ivoire	Secondary Education	1980	3.774
Côte d'Ivoire	Secondary Education	1985	3.764
Côte d'Ivoire	Higher Education	1955	3.950
Côte d'Ivoire	Higher Education	1960	3.500
Côte d'Ivoire	Higher Education	1965	3.250
Côte d'Ivoire	Higher Education	1970	3.150
Côte d'Ivoire	Higher Education	1975	3.050
Côte d'Ivoire	Higher Education	1980	3.121
Côte d'Ivoire	Higher Education	1985	3.140
Democratic Republic of Congo	No Education	1955	7.100
Democratic Republic of Congo	No Education	1960	7.150
Democratic Republic of Congo	No Education	1965	7.300
Democratic Republic of Congo	No Education	1970	7.250
Democratic Republic of Congo	No Education	1975	7.150
Democratic Republic of Congo	No Education	1980	7.082
Democratic Republic of Congo	No Education	1985	7.249
Democratic Republic of Congo	Secondary Education	1955	5.250
Democratic Republic of Congo	Secondary Education	1960	4.950
Democratic Republic of Congo	Secondary Education	1965	4.950
Democratic Republic of Congo	Secondary Education	1970	5.000
Democratic Republic of Congo	Secondary Education	1975	5.100
Democratic Republic of Congo	Secondary Education	1980	5.066
Democratic Republic of Congo	Secondary Education	1985	5.281
Democratic Republic of Congo	Higher Education	1955	4.250
Democratic Republic of Congo	Higher Education	1960	4.000
Democratic Republic of Congo	Higher Education	1965	4.000
Democratic Republic of Congo	Higher Education	1970	3.850
Democratic Republic of Congo	Higher Education	1975	3.850
Democratic Republic of Congo	Higher Education	1980	3.674
Country	Education	Cohort	TFR
Democratic Republic of Congo	Higher Education	1985	3.929
Egypt	No Education	1955	5.350

Egypt	No Education	1960	4.900
Egypt	No Education	1965	4.600
Egypt	No Education	1970	4.350
Egypt	No Education	1975	4.200
Egypt	No Education	1980	4.038
Egypt	No Education	1985	3.960
Egypt	Secondary Education	1955	4.050
Egypt	Secondary Education	1960	3.700
Egypt	Secondary Education	1965	3.350
Egypt	Secondary Education	1970	3.100
Egypt	Secondary Education	1975	2.850
Egypt	Secondary Education	1980	2.844
Egypt	Secondary Education	1985	2.906
Egypt	Higher Education	1955	3.200
Egypt	Higher Education	1960	2.950
Egypt	Higher Education	1965	2.700
Egypt	Higher Education	1970	2.600
Egypt	Higher Education	1975	2.350
Egypt	Higher Education	1980	2.308
Egypt	Higher Education	1985	2.287
Eswatini	No Education	1955	6.500
Eswatini	No Education	1960	6.450
Eswatini	No Education	1965	6.050
Eswatini	No Education	1970	5.400
Eswatini	No Education	1975	4.900
Eswatini	No Education	1980	4.268
Eswatini	No Education	1985	3.538
Eswatini	Secondary Education	1955	4.400
Eswatini	Secondary Education	1960	4.250
Eswatini	Secondary Education	1965	3.900
Eswatini	Secondary Education	1970	3.550
Eswatini	Secondary Education	1975	3.200
Eswatini	Secondary Education	1980	3.085
Eswatini	Secondary Education	1985	2.898
Eswatini	Higher Education	1955	3.600
Eswatini	Higher Education	1960	3.350
Eswatini	Higher Education	1965	3.050
Eswatini	Higher Education	1970	2.750
Country	Education	Cohort	TFR
Eswatini	Higher Education	1975	2.550
Eswatini	Higher Education	1980	2.231

Eswatini	Higher Education	1985	2.117
Ethiopia	No Education	1955	6.750
Ethiopia	No Education	1960	6.750
Ethiopia	No Education	1965	6.650
Ethiopia	No Education	1970	6.350
Ethiopia	No Education	1975	6.000
Ethiopia	No Education	1980	5.325
Ethiopia	No Education	1985	4.559
Ethiopia	Secondary Education	1955	4.250
Ethiopia	Secondary Education	1960	3.950
Ethiopia	Secondary Education	1965	3.800
Ethiopia	Secondary Education	1970	3.750
Ethiopia	Secondary Education	1975	3.600
Ethiopia	Secondary Education	1980	3.466
Ethiopia	Secondary Education	1985	3.375
Ethiopia	Higher Education	1955	3.350
Ethiopia	Higher Education	1960	3.000
Ethiopia	Higher Education	1965	2.950
Ethiopia	Higher Education	1970	2.850
Ethiopia	Higher Education	1975	2.750
Ethiopia	Higher Education	1980	2.630
Ethiopia	Higher Education	1985	2.488
Gabon	No Education	1955	6.750
Gabon	No Education	1960	6.300
Gabon	No Education	1965	5.900
Gabon	No Education	1970	5.450
Gabon	No Education	1975	5.400
Gabon	No Education	1980	5.232
Gabon	No Education	1985	5.425
Gabon	Secondary Education	1955	4.450
Gabon	Secondary Education	1960	4.200
Gabon	Secondary Education	1965	3.950
Gabon	Secondary Education	1970	3.800
Gabon	Secondary Education	1975	3.600
Gabon	Secondary Education	1980	3.435
Gabon	Secondary Education	1985	3.340
Gabon	Higher Education	1955	3.600
Gabon	Higher Education	1960	3.250
Country	Education	Cohort	TFR
Gabon	Higher Education	1965	3.050
Gabon	Higher Education	1970	3.000

Gabon	Higher Education	1975	2.850
Gabon	Higher Education	1980	2.864
Gabon	Higher Education	1985	3.004
Gambia	No Education	1955	6.500
Gambia	No Education	1960	6.450
Gambia	No Education	1965	6.300
Gambia	No Education	1970	6.100
Gambia	No Education	1975	6.050
Gambia	No Education	1980	6.028
Gambia	No Education	1985	5.819
Gambia	Secondary Education	1955	5.750
Gambia	Secondary Education	1960	5.300
Gambia	Secondary Education	1965	4.750
Gambia	Secondary Education	1970	4.400
Gambia	Secondary Education	1975	4.200
Gambia	Secondary Education	1980	4.171
Gambia	Secondary Education	1985	4.017
Gambia	Higher Education	1955	4.650
Gambia	Higher Education	1960	4.050
Gambia	Higher Education	1965	3.750
Gambia	Higher Education	1970	3.500
Gambia	Higher Education	1975	3.350
Gambia	Higher Education	1980	3.393
Gambia	Higher Education	1985	3.308
Ghana	No Education	1955	6.600
Ghana	No Education	1960	6.300
Ghana	No Education	1965	5.850
Ghana	No Education	1970	5.450
Ghana	No Education	1975	5.200
Ghana	No Education	1980	5.077
Ghana	No Education	1985	4.968
Ghana	Secondary Education	1955	4.500
Ghana	Secondary Education	1960	4.150
Ghana	Secondary Education	1965	3.950
Ghana	Secondary Education	1970	3.650
Ghana	Secondary Education	1975	3.450
Ghana	Secondary Education	1980	3.339
Ghana	Secondary Education	1985	3.225
Country	Education	Cohort	TFR
Ghana	Higher Education	1955	3.550
Ghana	Higher Education	1960	3.350

Ghana	Higher Education	1965	3.150
Ghana	Higher Education	1970	3.050
Ghana	Higher Education	1975	3.000
Ghana	Higher Education	1980	2.855
Ghana	Higher Education	1985	2.774
Guinea	No Education	1955	6.250
Guinea	No Education	1960	6.250
Guinea	No Education	1965	6.150
Guinea	No Education	1970	5.850
Guinea	No Education	1975	5.500
Guinea	No Education	1980	5.393
Guinea	No Education	1985	5.135
Guinea	Secondary Education	1955	4.900
Guinea	Secondary Education	1960	4.600
Guinea	Secondary Education	1965	4.400
Guinea	Secondary Education	1970	4.100
Guinea	Secondary Education	1975	4.000
Guinea	Secondary Education	1980	3.752
Guinea	Secondary Education	1985	3.693
Guinea	Higher Education	1955	4.050
Guinea	Higher Education	1960	3.650
Guinea	Higher Education	1965	3.550
Guinea	Higher Education	1970	3.150
Guinea	Higher Education	1975	3.050
Guinea	Higher Education	1980	2.942
Guinea	Higher Education	1985	2.919
Kenya	No Education	1955	7.150
Kenya	No Education	1960	7.000
Kenya	No Education	1965	6.750
Kenya	No Education	1970	6.250
Kenya	No Education	1975	5.900
Kenya	No Education	1980	5.513
Kenya	No Education	1985	5.365
Kenya	Secondary Education	1955	5.000
Kenya	Secondary Education	1960	4.650
Kenya	Secondary Education	1965	4.400
Kenya	Secondary Education	1970	4.050
Kenya	Secondary Education	1975	3.800
Country	Education	Cohort	TFR
Kenya	Secondary Education	1980	3.510
Kenya	Secondary Education	1985	3.276

Kenya	Higher Education	1955	4.000
Kenya	Higher Education	1960	3.700
Kenya	Higher Education	1965	3.500
Kenya	Higher Education	1970	3.250
Kenya	Higher Education	1975	3.200
Kenya	Higher Education	1980	2.977
Kenya	Higher Education	1985	2.806
Lesotho	No Education	1955	5.650
Lesotho	No Education	1960	5.500
Lesotho	No Education	1965	5.350
Lesotho	No Education	1970	4.850
Lesotho	No Education	1975	4.500
Lesotho	No Education	1980	3.906
Lesotho	No Education	1985	3.543
Lesotho	Secondary Education	1955	4.000
Lesotho	Secondary Education	1960	3.800
Lesotho	Secondary Education	1965	3.500
Lesotho	Secondary Education	1970	3.200
Lesotho	Secondary Education	1975	2.850
Lesotho	Secondary Education	1980	2.617
Lesotho	Secondary Education	1985	2.601
Lesotho	Higher Education	1955	3.050
Lesotho	Higher Education	1960	2.900
Lesotho	Higher Education	1965	2.800
Lesotho	Higher Education	1970	2.450
Lesotho	Higher Education	1975	2.400
Lesotho	Higher Education	1980	2.011
Lesotho	Higher Education	1985	1.813
Liberia	No Education	1955	6.450
Liberia	No Education	1960	6.350
Liberia	No Education	1965	6.200
Liberia	No Education	1970	5.800
Liberia	No Education	1975	5.450
Liberia	No Education	1980	5.187
Liberia	No Education	1985	4.469
Liberia	Secondary Education	1955	4.800
Liberia	Secondary Education	1960	4.650
Liberia	Secondary Education	1965	4.450
Country	Education	Cohort	TFR
Liberia	Secondary Education	1970	4.350
Liberia	Secondary Education	1975	4.050

Liberia	Secondary Education	1980	3.827
Liberia	Secondary Education	1985	3.524
Liberia	Higher Education	1955	3.850
Liberia	Higher Education	1960	3.700
Liberia	Higher Education	1965	3.600
Liberia	Higher Education	1970	3.350
Liberia	Higher Education	1975	3.200
Liberia	Higher Education	1980	2.956
Liberia	Higher Education	1985	2.684
Madagascar	No Education	1955	7.000
Madagascar	No Education	1960	6.350
Madagascar	No Education	1965	6.300
Madagascar	No Education	1970	5.950
Madagascar	No Education	1975	5.800
Madagascar	No Education	1980	5.319
Madagascar	No Education	1985	4.738
Madagascar	Secondary Education	1955	4.550
Madagascar	Secondary Education	1960	4.200
Madagascar	Secondary Education	1965	4.200
Madagascar	Secondary Education	1970	4.100
Madagascar	Secondary Education	1975	4.000
Madagascar	Secondary Education	1980	3.685
Madagascar	Secondary Education	1985	3.143
Madagascar	Higher Education	1955	3.550
Madagascar	Higher Education	1960	3.450
Madagascar	Higher Education	1965	3.300
Madagascar	Higher Education	1970	3.200
Madagascar	Higher Education	1975	3.050
Madagascar	Higher Education	1980	2.880
Madagascar	Higher Education	1985	2.564
Malawi	No Education	1955	7.000
Malawi	No Education	1960	6.700
Malawi	No Education	1965	6.500
Malawi	No Education	1970	6.350
Malawi	No Education	1975	5.950
Malawi	No Education	1980	5.538
Malawi	No Education	1985	5.235
Malawi	Secondary Education	1955	5.100
Country	Education	Cohort	TFR
Malawi	Secondary Education	1960	4.650
Malawi	Secondary Education	1965	4.400

Malawi	Secondary Education	1970	4.250
Malawi	Secondary Education	1975	4.100
Malawi	Secondary Education	1980	3.723
Malawi	Secondary Education	1985	3.622
Malawi	Higher Education	1955	4.150
Malawi	Higher Education	1960	3.850
Malawi	Higher Education	1965	3.600
Malawi	Higher Education	1970	3.350
Malawi	Higher Education	1975	3.200
Malawi	Higher Education	1980	3.049
Malawi	Higher Education	1985	2.915
Mali	No Education	1955	7.200
Mali	No Education	1960	7.050
Mali	No Education	1965	6.900
Mali	No Education	1970	6.700
Mali	No Education	1975	6.500
Mali	No Education	1980	6.443
Mali	No Education	1985	6.478
Mali	Secondary Education	1955	5.200
Mali	Secondary Education	1960	4.800
Mali	Secondary Education	1965	4.700
Mali	Secondary Education	1970	4.450
Mali	Secondary Education	1975	4.400
Mali	Secondary Education	1980	4.343
Mali	Secondary Education	1985	4.488
Mali	Higher Education	1955	4.100
Mali	Higher Education	1960	3.850
Mali	Higher Education	1965	3.650
Mali	Higher Education	1970	3.450
Mali	Higher Education	1975	3.500
Mali	Higher Education	1980	3.464
Mali	Higher Education	1985	3.614
Morocco	No Education	1955	5.050
Morocco	No Education	1960	4.350
Morocco	No Education	1965	3.550
Morocco	No Education	1970	3.050
Morocco	No Education	1975	2.800
Morocco	No Education	1980	2.639
Country	Education	Cohort	TFR
Morocco	No Education	1985	2.471
Morocco	Secondary Education	1955	3.250

Morocco	Secondary Education	1960	2.800
Morocco	Secondary Education	1965	2.650
Morocco	Secondary Education	1970	2.600
Morocco	Secondary Education	1975	2.450
Morocco	Secondary Education	1980	2.378
Morocco	Secondary Education	1985	2.371
Morocco	Higher Education	1955	2.600
Morocco	Higher Education	1960	2.300
Morocco	Higher Education	1965	2.200
Morocco	Higher Education	1970	2.150
Morocco	Higher Education	1975	2.200
Morocco	Higher Education	1980	2.235
Morocco	Higher Education	1985	2.219
Mozambique	No Education	1955	6.100
Mozambique	No Education	1960	6.050
Mozambique	No Education	1965	5.750
Mozambique	No Education	1970	5.700
Mozambique	No Education	1975	5.550
Mozambique	No Education	1980	5.505
Mozambique	No Education	1985	5.471
Mozambique	Secondary Education	1955	4.450
Mozambique	Secondary Education	1960	4.250
Mozambique	Secondary Education	1965	3.900
Mozambique	Secondary Education	1970	3.750
Mozambique	Secondary Education	1975	3.550
Mozambique	Secondary Education	1980	3.404
Mozambique	Secondary Education	1985	3.322
Mozambique	Higher Education	1955	3.450
Mozambique	Higher Education	1960	3.300
Mozambique	Higher Education	1965	3.100
Mozambique	Higher Education	1970	2.950
Mozambique	Higher Education	1975	2.900
Mozambique	Higher Education	1980	2.812
Mozambique	Higher Education	1985	2.646
Namibia	No Education	1955	6.100
Namibia	No Education	1960	5.600
Namibia	No Education	1965	5.400
Namibia	No Education	1970	5.100
Country	Education	Cohort	TFR
Namibia	No Education	1975	5.050
Namibia	No Education	1980	4.846

Namibia	No Education	1985	4.713
Namibia	Secondary Education	1955	4.050
Namibia	Secondary Education	1960	3.850
Namibia	Secondary Education	1965	3.550
Namibia	Secondary Education	1970	3.250
Namibia	Secondary Education	1975	3.100
Namibia	Secondary Education	1980	3.080
Namibia	Secondary Education	1985	3.044
Namibia	Higher Education	1955	3.300
Namibia	Higher Education	1960	2.950
Namibia	Higher Education	1965	2.800
Namibia	Higher Education	1970	2.700
Namibia	Higher Education	1975	2.650
Namibia	Higher Education	1980	2.483
Namibia	Higher Education	1985	2.496
Niger	No Education	1955	7.400
Niger	No Education	1960	7.600
Niger	No Education	1965	7.700
Niger	No Education	1970	7.550
Niger	No Education	1975	7.350
Niger	No Education	1980	7.293
Niger	No Education	1985	7.329
Niger	Secondary Education	1955	5.500
Niger	Secondary Education	1960	5.150
Niger	Secondary Education	1965	5.000
Niger	Secondary Education	1970	5.000
Niger	Secondary Education	1975	4.950
Niger	Secondary Education	1980	4.543
Niger	Secondary Education	1985	4.331
Niger	Higher Education	1955	4.400
Niger	Higher Education	1960	4.150
Niger	Higher Education	1965	4.050
Niger	Higher Education	1970	3.850
Niger	Higher Education	1975	3.700
Niger	Higher Education	1980	3.369
Niger	Higher Education	1985	2.774
Nigeria	No Education	1955	6.450
Nigeria	No Education	1960	6.750
Country	Education	Cohort	TFR
Nigeria	No Education	1965	6.650
Nigeria	No Education	1970	6.650

Nigeria	No Education	1975	6.500
Nigeria	No Education	1980	6.511
Nigeria	No Education	1985	6.541
Nigeria	Secondary Education	1955	5.200
Nigeria	Secondary Education	1960	5.000
Nigeria	Secondary Education	1965	4.800
Nigeria	Secondary Education	1970	4.600
Nigeria	Secondary Education	1975	4.650
Nigeria	Secondary Education	1980	4.439
Nigeria	Secondary Education	1985	4.476
Nigeria	Higher Education	1955	4.200
Nigeria	Higher Education	1960	3.850
Nigeria	Higher Education	1965	3.650
Nigeria	Higher Education	1970	3.650
Nigeria	Higher Education	1975	3.600
Nigeria	Higher Education	1980	3.463
Nigeria	Higher Education	1985	3.543
Rwanda	No Education	1955	7.400
Rwanda	No Education	1960	6.850
Rwanda	No Education	1965	6.500
Rwanda	No Education	1970	6.150
Rwanda	No Education	1975	5.850
Rwanda	No Education	1980	5.284
Rwanda	No Education	1985	4.762
Rwanda	Secondary Education	1955	5.200
Rwanda	Secondary Education	1960	4.800
Rwanda	Secondary Education	1965	4.600
Rwanda	Secondary Education	1970	4.350
Rwanda	Secondary Education	1975	4.200
Rwanda	Secondary Education	1980	3.671
Rwanda	Secondary Education	1985	3.009
Rwanda	Higher Education	1955	4.150
Rwanda	Higher Education	1960	4.000
Rwanda	Higher Education	1965	3.850
Rwanda	Higher Education	1970	3.550
Rwanda	Higher Education	1975	3.300
Rwanda	Higher Education	1980	2.946
Rwanda	Higher Education	1985	2.483
Country	Education	Cohort	TFR
Sao Tome and Principe	No Education	1955	7.350
Sao Tome and Principe	No Education	1960	7.300

Sao Tome and Principe	No Education	1965	7.200
Sao Tome and Principe	No Education	1970	6.350
Sao Tome and Principe	No Education	1975	5.600
Sao Tome and Principe	No Education	1980	4.940
Sao Tome and Principe	No Education	1985	4.340
Sao Tome and Principe	Secondary Education	1955	4.650
Sao Tome and Principe	Secondary Education	1960	5.100
Sao Tome and Principe	Secondary Education	1965	4.900
Sao Tome and Principe	Secondary Education	1970	4.300
Sao Tome and Principe	Secondary Education	1975	3.750
Sao Tome and Principe	Secondary Education	1980	3.497
Sao Tome and Principe	Secondary Education	1985	3.018
Sao Tome and Principe	Higher Education	1955	3.900
Sao Tome and Principe	Higher Education	1960	3.650
Sao Tome and Principe	Higher Education	1965	3.700
Sao Tome and Principe	Higher Education	1970	3.450
Sao Tome and Principe	Higher Education	1975	2.950
Sao Tome and Principe	Higher Education	1980	2.625
Sao Tome and Principe	Higher Education	1985	2.327
Senegal	No Education	1955	7.050
Senegal	No Education	1960	6.650
Senegal	No Education	1965	6.250
Senegal	No Education	1970	5.850
Senegal	No Education	1975	5.500
Senegal	No Education	1980	5.313
Senegal	No Education	1985	5.299
Senegal	Secondary Education	1955	4.550
Senegal	Secondary Education	1960	4.150
Senegal	Secondary Education	1965	3.900
Senegal	Secondary Education	1970	3.750
Senegal	Secondary Education	1975	3.600
Senegal	Secondary Education	1980	3.559
Senegal	Secondary Education	1985	3.629
Senegal	Higher Education	1955	3.650
Senegal	Higher Education	1960	3.250
Senegal	Higher Education	1965	3.000
Senegal	Higher Education	1970	3.000
Senegal	Higher Education	1975	2.800
Country	Education	Cohort	TFR
Senegal	Higher Education	1980	2.921
Senegal	Higher Education	1985	2.939

Sierra Leone	No Education	1955	6.800
Sierra Leone	No Education	1960	6.650
Sierra Leone	No Education	1965	6.400
Sierra Leone	No Education	1970	6.050
Sierra Leone	No Education	1975	5.600
Sierra Leone	No Education	1980	5.273
Sierra Leone	No Education	1985	5.057
Sierra Leone	Secondary Education	1955	4.950
Sierra Leone	Secondary Education	1960	4.500
Sierra Leone	Secondary Education	1965	4.300
Sierra Leone	Secondary Education	1970	4.000
Sierra Leone	Secondary Education	1975	3.900
Sierra Leone	Secondary Education	1980	3.567
Sierra Leone	Secondary Education	1985	3.338
Sierra Leone	Higher Education	1955	3.900
Sierra Leone	Higher Education	1960	3.550
Sierra Leone	Higher Education	1965	3.300
Sierra Leone	Higher Education	1970	3.150
Sierra Leone	Higher Education	1975	3.050
Sierra Leone	Higher Education	1980	2.991
Sierra Leone	Higher Education	1985	2.951
South Africa	No Education	1955	4.900
South Africa	No Education	1960	4.650
South Africa	No Education	1965	4.400
South Africa	No Education	1970	4.100
South Africa	No Education	1975	3.900
South Africa	No Education	1980	3.696
South Africa	No Education	1985	3.576
South Africa	Secondary Education	1955	3.250
South Africa	Secondary Education	1960	3.150
South Africa	Secondary Education	1965	2.750
South Africa	Secondary Education	1970	2.450
South Africa	Secondary Education	1975	2.250
South Africa	Secondary Education	1980	2.175
South Africa	Secondary Education	1985	2.157
South Africa	Higher Education	1955	2.600
South Africa	Higher Education	1960	2.450
South Africa	Higher Education	1965	2.250
Country	Education	Cohort	TFR
South Africa	Higher Education	1970	2.150
South Africa	Higher Education	1975	2.100

South Africa	Higher Education	1980	1.950
South Africa	Higher Education	1985	1.934
Sudan	No Education	1955	6.750
Sudan	No Education	1960	6.100
Sudan	No Education	1965	5.600
Sudan	No Education	1970	5.000
Sudan	No Education	1975	4.650
Sudan	No Education	1980	4.875
Sudan	No Education	1985	5.312
Sudan	Secondary Education	1955	5.000
Sudan	Secondary Education	1960	5.000
Sudan	Secondary Education	1965	5.150
Sudan	Secondary Education	1970	5.150
Sudan	Secondary Education	1975	4.900
Sudan	Secondary Education	1980	4.288
Sudan	Secondary Education	1985	4.304
Sudan	Higher Education	1955	4.100
Sudan	Higher Education	1960	4.200
Sudan	Higher Education	1965	4.450
Sudan	Higher Education	1970	4.450
Sudan	Higher Education	1975	4.150
Sudan	Higher Education	1980	3.643
Sudan	Higher Education	1985	3.883
Tanzania	No Education	1955	6.800
Tanzania	No Education	1960	6.450
Tanzania	No Education	1965	6.300
Tanzania	No Education	1970	6.150
Tanzania	No Education	1975	5.950
Tanzania	No Education	1980	5.657
Tanzania	No Education	1985	5.616
Tanzania	Secondary Education	1955	4.650
Tanzania	Secondary Education	1960	4.350
Tanzania	Secondary Education	1965	4.150
Tanzania	Secondary Education	1970	4.050
Tanzania	Secondary Education	1975	3.950
Tanzania	Secondary Education	1980	3.900
Tanzania	Secondary Education	1985	3.863
Tanzania	Higher Education	1955	3.700
Country	Education	Cohort	TFR
Tanzania	Higher Education	1960	3.500
Tanzania	Higher Education	1965	3.350

Tanzania	Higher Education	1970	3.300
Tanzania	Higher Education	1975	3.100
Tanzania	Higher Education	1980	2.975
Tanzania	Higher Education	1985	2.746
Togo	No Education	1955	6.150
Togo	No Education	1960	5.950
Togo	No Education	1965	5.750
Togo	No Education	1970	5.550
Togo	No Education	1975	5.200
Togo	No Education	1980	5.082
Togo	No Education	1985	5.038
Togo	Secondary Education	1955	4.650
Togo	Secondary Education	1960	4.150
Togo	Secondary Education	1965	3.950
Togo	Secondary Education	1970	3.750
Togo	Secondary Education	1975	3.650
Togo	Secondary Education	1980	3.667
Togo	Secondary Education	1985	3.654
Togo	Higher Education	1955	3.750
Togo	Higher Education	1960	3.250
Togo	Higher Education	1965	3.100
Togo	Higher Education	1970	3.000
Togo	Higher Education	1975	3.000
Togo	Higher Education	1980	2.910
Togo	Higher Education	1985	2.906
Tunisia	No Education	1955	4.450
Tunisia	No Education	1960	3.750
Tunisia	No Education	1965	3.450
Tunisia	No Education	1970	3.000
Tunisia	No Education	1975	3.550
Tunisia	No Education	1980	4.038
Tunisia	No Education	1985	4.205
Tunisia	Secondary Education	1955	3.700
Tunisia	Secondary Education	1960	3.250
Tunisia	Secondary Education	1965	2.800
Tunisia	Secondary Education	1970	2.150
Tunisia	Secondary Education	1975	2.050
Tunisia	Secondary Education	1980	1.687
Country	Education	Cohort	TFR
Tunisia	Secondary Education	1985	1.662
Tunisia	Higher Education	1955	2.950

Tunisia	Higher Education	1960	2.650
Tunisia	Higher Education	1965	2.450
Tunisia	Higher Education	1970	2.350
Tunisia	Higher Education	1975	2.350
Tunisia	Higher Education	1980	2.255
Tunisia	Higher Education	1985	2.336
Uganda	No Education	1955	7.350
Uganda	No Education	1960	7.450
Uganda	No Education	1965	7.450
Uganda	No Education	1970	7.250
Uganda	No Education	1975	7.150
Uganda	No Education	1980	6.814
Uganda	No Education	1985	6.800
Uganda	Secondary Education	1955	5.900
Uganda	Secondary Education	1960	5.500
Uganda	Secondary Education	1965	5.400
Uganda	Secondary Education	1970	5.100
Uganda	Secondary Education	1975	4.950
Uganda	Secondary Education	1980	4.602
Uganda	Secondary Education	1985	4.370
Uganda	Higher Education	1955	4.600
Uganda	Higher Education	1960	4.300
Uganda	Higher Education	1965	4.150
Uganda	Higher Education	1970	3.950
Uganda	Higher Education	1975	3.900
Uganda	Higher Education	1980	3.640
Uganda	Higher Education	1985	3.522
Zambia	No Education	1955	7.600
Zambia	No Education	1960	7.250
Zambia	No Education	1965	7.050
Zambia	No Education	1970	6.850
Zambia	No Education	1975	6.650
Zambia	No Education	1980	6.491
Zambia	No Education	1985	6.489
Zambia	Secondary Education	1955	5.250
Zambia	Secondary Education	1960	4.850
Zambia	Secondary Education	1965	4.750
Zambia	Secondary Education	1970	4.500
Country	Education	Cohort	TFR
Zambia	Secondary Education	1975	4.450
Zambia	Secondary Education	1980	4.253

Zambia	Secondary Education	1985	4.241
Zambia	Higher Education	1955	4.250
Zambia	Higher Education	1960	3.850
Zambia	Higher Education	1965	3.700
Zambia	Higher Education	1970	3.550
Zambia	Higher Education	1975	3.500
Zambia	Higher Education	1980	3.300
Zambia	Higher Education	1985	3.314
Zimbabwe	No Education	1955	6.750
Zimbabwe	No Education	1960	6.350
Zimbabwe	No Education	1965	5.950
Zimbabwe	No Education	1970	5.500
Zimbabwe	No Education	1975	5.200
Zimbabwe	No Education	1980	5.117
Zimbabwe	No Education	1985	4.940
Zimbabwe	Secondary Education	1955	4.450
Zimbabwe	Secondary Education	1960	4.050
Zimbabwe	Secondary Education	1965	3.650
Zimbabwe	Secondary Education	1970	3.350
Zimbabwe	Secondary Education	1975	3.250
Zimbabwe	Secondary Education	1980	3.316
Zimbabwe	Secondary Education	1985	3.474
Zimbabwe	Higher Education	1955	3.600
Zimbabwe	Higher Education	1960	3.300
Zimbabwe	Higher Education	1965	3.000
Zimbabwe	Higher Education	1970	2.750
Zimbabwe	Higher Education	1975	2.700
Zimbabwe	Higher Education	1980	2.547
Zimbabwe	Higher Education	1985	2.716
Angola	No Education	1985	6.084
Angola	No Education	1990	5.957
Angola	No Education	1995	5.522
Angola	No Education	2000	4.986
Angola	Secondary Education	1985	4.366
Angola	Secondary Education	1990	4.263
Angola	Secondary Education	1995	3.910
Angola	Secondary Education	2000	3.479
Angola	Higher Education	1985	3.607
Country	Education	Cohort	TFR
Angola	Higher Education	1990	3.521
Angola	Higher Education	1995	3.227

Angola	Higher Education	2000	2.870
Benin	No Education	1985	5.614
Benin	No Education	1990	5.260
Benin	No Education	1995	4.755
Benin	No Education	2000	4.847
Benin	Secondary Education	1985	3.605
Benin	Secondary Education	1990	3.347
Benin	Secondary Education	1995	2.983
Benin	Secondary Education	2000	3.048
Benin	Higher Education	1985	2.844
Benin	Higher Education	1990	2.639
Benin	Higher Education	1995	2.350
Benin	Higher Education	2000	2.402
Burkina Faso	No Education	1985	5.508
Burkina Faso	No Education	1990	5.532
Burkina Faso	No Education	1995	5.328
Burkina Faso	No Education	2000	5.231
Burkina Faso	Secondary Education	1985	3.655
Burkina Faso	Secondary Education	1990	3.673
Burkina Faso	Secondary Education	1995	3.519
Burkina Faso	Secondary Education	2000	3.447
Burkina Faso	Higher Education	1985	2.971
Burkina Faso	Higher Education	1990	2.985
Burkina Faso	Higher Education	1995	2.860
Burkina Faso	Higher Education	2000	2.800
Burundi	No Education	1985	5.729
Burundi	No Education	1990	5.625
Burundi	No Education	1995	4.795
Burundi	No Education	2000	4.380
Burundi	Secondary Education	1985	4.291
Burundi	Secondary Education	1990	4.202
Burundi	Secondary Education	1995	3.499
Burundi	Secondary Education	2000	3.155
Burundi	Higher Education	1985	3.386
Burundi	Higher Education	1990	3.316
Burundi	Higher Education	1995	2.758
Burundi	Higher Education	2000	2.485
Cameroon	No Education	1985	5.739
Country	Education	Cohort	TFR
Cameroon	No Education	1990	4.995
Cameroon	No Education	1995	4.616

Cameroon	No Education	2000	4.640
Cameroon	Secondary Education	1985	4.169
Cameroon	Secondary Education	1990	3.556
Cameroon	Secondary Education	1995	3.250
Cameroon	Secondary Education	2000	3.269
Cameroon	Higher Education	1985	3.235
Cameroon	Higher Education	1990	2.757
Cameroon	Higher Education	1995	2.518
Cameroon	Higher Education	2000	2.533
Central African Republic	No Education	1985	5.793
Central African Republic	No Education	1990	5.429
Central African Republic	No Education	1995	5.607
Central African Republic	No Education	2000	5.480
Central African Republic	Secondary Education	1985	4.069
Central African Republic	Secondary Education	1990	3.778
Central African Republic	Secondary Education	1995	3.919
Central African Republic	Secondary Education	2000	3.818
Central African Republic	Higher Education	1985	2.928
Central African Republic	Higher Education	1990	2.718
Central African Republic	Higher Education	1995	2.820
Central African Republic	Higher Education	2000	2.747
Chad	No Education	1985	6.941
Chad	No Education	1990	7.008
Chad	No Education	1995	6.218
Chad	No Education	2000	6.335
Chad	Secondary Education	1985	4.926
Chad	Secondary Education	1990	4.981
Chad	Secondary Education	1995	4.344
Chad	Secondary Education	2000	4.436
Chad	Higher Education	1985	3.580
Chad	Higher Education	1990	3.620
Chad	Higher Education	1995	3.154
Chad	Higher Education	2000	3.222
Comoros	No Education	1985	5.487
Comoros	No Education	1990	5.232
Comoros	No Education	1995	4.377
Comoros	No Education	2000	4.811
Comoros	Secondary Education	1985	4.066
Country	Education	Cohort	TFR
Comoros	Secondary Education	1990	3.852
Comoros	Secondary Education	1995	3.138

Comoros	Secondary Education	2000	3.491
Comoros	Higher Education	1985	3.369
Comoros	Higher Education	1990	3.191
Comoros	Higher Education	1995	2.596
Comoros	Higher Education	2000	2.889
Congo	No Education	1985	5.663
Congo	No Education	1990	4.550
Congo	No Education	1995	4.310
Congo	No Education	2000	4.784
Congo	Secondary Education	1985	3.755
Congo	Secondary Education	1990	2.918
Congo	Secondary Education	1995	2.743
Congo	Secondary Education	2000	3.085
Congo	Higher Education	1985	2.835
Congo	Higher Education	1990	2.199
Congo	Higher Education	1995	2.067
Congo	Higher Education	2000	2.326
Egypt	No Education	1985	3.960
Egypt	No Education	1990	3.441
Egypt	No Education	1995	2.970
Egypt	No Education	2000	2.553
Egypt	Secondary Education	1985	2.906
Egypt	Secondary Education	1990	2.474
Egypt	Secondary Education	1995	2.090
Egypt	Secondary Education	2000	1.757
Egypt	Higher Education	1985	2.287
Egypt	Higher Education	1990	1.945
Egypt	Higher Education	1995	1.642
Egypt	Higher Education	2000	1.378
Ethiopia	No Education	1985	4.559
Ethiopia	No Education	1990	4.260
Ethiopia	No Education	1995	3.996
Ethiopia	No Education	2000	3.825
Ethiopia	Secondary Education	1985	3.375
Ethiopia	Secondary Education	1990	3.124
Ethiopia	Secondary Education	1995	2.904
Ethiopia	Secondary Education	2000	2.763
Ethiopia	Higher Education	1985	2.488
Country	Education	Cohort	TFR
Ethiopia	Higher Education	1990	2.302
Ethiopia	Higher Education	1995	2.139

Ethiopia	Higher Education	2000	2.035
Gabon	No Education	1985	5.425
Gabon	No Education	1990	5.171
Gabon	No Education	1995	3.840
Gabon	No Education	2000	4.235
Gabon	Secondary Education	1985	3.340
Gabon	Secondary Education	1990	3.163
Gabon	Secondary Education	1995	2.239
Gabon	Secondary Education	2000	2.500
Gabon	Higher Education	1985	3.004
Gabon	Higher Education	1990	2.843
Gabon	Higher Education	1995	2.008
Gabon	Higher Education	2000	2.243
Gambia	No Education	1985	5.819
Gambia	No Education	1990	5.380
Gambia	No Education	1995	5.077
Gambia	No Education	2000	4.503
Gambia	Secondary Education	1985	4.017
Gambia	Secondary Education	1990	3.674
Gambia	Secondary Education	1995	3.439
Gambia	Secondary Education	2000	2.999
Gambia	Higher Education	1985	3.308
Gambia	Higher Education	1990	3.023
Gambia	Higher Education	1995	2.829
Gambia	Higher Education	2000	2.464
Ghana	No Education	1985	4.968
Ghana	No Education	1990	4.983
Ghana	No Education	1995	4.925
Ghana	No Education	2000	4.844
Ghana	Secondary Education	1985	3.225
Ghana	Secondary Education	1990	3.237
Ghana	Secondary Education	1995	3.194
Ghana	Secondary Education	2000	3.134
Ghana	Higher Education	1985	2.774
Ghana	Higher Education	1990	2.784
Ghana	Higher Education	1995	2.747
Ghana	Higher Education	2000	2.695
Guinea	No Education	1985	5.135
Country	Education	Cohort	TFR
Guinea	No Education	1990	5.066
Guinea	No Education	1995	4.707

Guinea	No Education	2000	4.657
Guinea	Secondary Education	1985	3.693
Guinea	Secondary Education	1990	3.637
Guinea	Secondary Education	1995	3.344
Guinea	Secondary Education	2000	3.304
Guinea	Higher Education	1985	2.919
Guinea	Higher Education	1990	2.874
Guinea	Higher Education	1995	2.642
Guinea	Higher Education	2000	2.610
Kenya	No Education	1985	5.365
Kenya	No Education	1990	5.257
Kenya	No Education	1995	5.171
Kenya	No Education	2000	4.884
Kenya	Secondary Education	1985	3.276
Kenya	Secondary Education	1990	3.201
Kenya	Secondary Education	1995	3.142
Kenya	Secondary Education	2000	2.944
Kenya	Higher Education	1985	2.806
Kenya	Higher Education	1990	2.741
Kenya	Higher Education	1995	2.690
Kenya	Higher Education	2000	2.520
Lesotho	No Education	1985	3.543
Lesotho	No Education	1990	3.056
Lesotho	No Education	1995	2.864
Lesotho	No Education	2000	2.712
Lesotho	Secondary Education	1985	2.601
Lesotho	Secondary Education	1990	2.196
Lesotho	Secondary Education	1995	2.039
Lesotho	Secondary Education	2000	1.917
Lesotho	Higher Education	1985	1.813
Lesotho	Higher Education	1990	1.529
Lesotho	Higher Education	1995	1.419
Lesotho	Higher Education	2000	1.334
Liberia	No Education	1985	4.469
Liberia	No Education	1990	4.245
Liberia	No Education	1995	4.354
Liberia	No Education	2000	3.844
Liberia	Secondary Education	1985	3.524
Country	Education	Cohort	TFR
Liberia	Secondary Education	1990	3.323
Liberia	Secondary Education	1995	3.420

Liberia	Secondary Education	2000	2.966
Liberia	Higher Education	1985	2.684
Liberia	Higher Education	1990	2.531
Liberia	Higher Education	1995	2.605
Liberia	Higher Education	2000	2.257
Madagascar	No Education	1985	4.738
Madagascar	No Education	1990	4.387
Madagascar	No Education	1995	4.253
Madagascar	No Education	2000	4.120
Madagascar	Secondary Education	1985	3.143
Madagascar	Secondary Education	1990	2.879
Madagascar	Secondary Education	1995	2.779
Madagascar	Secondary Education	2000	2.680
Madagascar	Higher Education	1985	2.564
Madagascar	Higher Education	1990	2.347
Madagascar	Higher Education	1995	2.265
Madagascar	Higher Education	2000	2.185
Malawi	No Education	1985	5.235
Malawi	No Education	1990	4.865
Malawi	No Education	1995	4.585
Malawi	No Education	2000	4.337
Malawi	Secondary Education	1985	3.622
Malawi	Secondary Education	1990	3.332
Malawi	Secondary Education	1995	3.114
Malawi	Secondary Education	2000	2.923
Malawi	Higher Education	1985	2.915
Malawi	Higher Education	1990	2.681
Malawi	Higher Education	1995	2.505
Malawi	Higher Education	2000	2.350
Mali	No Education	1985	6.478
Mali	No Education	1990	6.351
Mali	No Education	1995	6.211
Mali	No Education	2000	6.112
Mali	Secondary Education	1985	4.488
Mali	Secondary Education	1990	4.388
Mali	Secondary Education	1995	4.278
Mali	Secondary Education	2000	4.201
Mali	Higher Education	1985	3.614
Country	Education	Cohort	TFR
Mali	Higher Education	1990	3.533
Mali	Higher Education	1995	3.444

Mali	Higher Education	2000	3.381
Morocco	No Education	1985	2.471
Morocco	No Education	1990	2.350
Morocco	No Education	1995	1.947
Morocco	No Education	2000	1.721
Morocco	Secondary Education	1985	2.371
Morocco	Secondary Education	1990	2.239
Morocco	Secondary Education	1995	1.803
Morocco	Secondary Education	2000	1.566
Morocco	Higher Education	1985	2.219
Morocco	Higher Education	1990	2.095
Morocco	Higher Education	1995	1.684
Morocco	Higher Education	2000	1.461
Mozambique	No Education	1985	5.471
Mozambique	No Education	1990	5.080
Mozambique	No Education	1995	4.767
Mozambique	No Education	2000	4.650
Mozambique	Secondary Education	1985	3.322
Mozambique	Secondary Education	1990	3.052
Mozambique	Secondary Education	1995	2.839
Mozambique	Secondary Education	2000	2.759
Mozambique	Higher Education	1985	2.646
Mozambique	Higher Education	1990	2.430
Mozambique	Higher Education	1995	2.259
Mozambique	Higher Education	2000	2.196
Namibia	No Education	1985	4.713
Namibia	No Education	1990	4.469
Namibia	No Education	1995	4.661
Namibia	No Education	2000	3.993
Namibia	Secondary Education	1985	3.044
Namibia	Secondary Education	1990	2.865
Namibia	Secondary Education	1995	3.005
Namibia	Secondary Education	2000	2.516
Namibia	Higher Education	1985	2.496
Namibia	Higher Education	1990	2.348
Namibia	Higher Education	1995	2.463
Namibia	Higher Education	2000	2.060
Niger	No Education	1985	7.329
Country	Education	Cohort	TFR
Niger	No Education	1990	7.344
Niger	No Education	1995	7.095

Niger	No Education	2000	6.862
Niger	Secondary Education	1985	4.331
Niger	Secondary Education	1990	4.341
Niger	Secondary Education	1995	4.174
Niger	Secondary Education	2000	4.019
Niger	Higher Education	1985	2.774
Niger	Higher Education	1990	2.780
Niger	Higher Education	1995	2.673
Niger	Higher Education	2000	2.573
Nigeria	No Education	1985	6.541
Nigeria	No Education	1990	5.985
Nigeria	No Education	1995	5.798
Nigeria	No Education	2000	5.378
Nigeria	Secondary Education	1985	4.476
Nigeria	Secondary Education	1990	4.045
Nigeria	Secondary Education	1995	3.901
Nigeria	Secondary Education	2000	3.581
Nigeria	Higher Education	1985	3.543
Nigeria	Higher Education	1990	3.199
Nigeria	Higher Education	1995	3.085
Nigeria	Higher Education	2000	2.830
Rwanda	No Education	1985	4.762
Rwanda	No Education	1990	4.441
Rwanda	No Education	1995	4.046
Rwanda	No Education	2000	3.912
Rwanda	Secondary Education	1985	3.009
Rwanda	Secondary Education	1990	2.780
Rwanda	Secondary Education	1995	2.499
Rwanda	Secondary Education	2000	2.405
Rwanda	Higher Education	1985	2.483
Rwanda	Higher Education	1990	2.292
Rwanda	Higher Education	1995	2.059
Rwanda	Higher Education	2000	1.981
Sao Tome and Principe	No Education	1985	4.340
Sao Tome and Principe	No Education	1990	4.419
Sao Tome and Principe	No Education	1995	3.301
Sao Tome and Principe	No Education	2000	3.305
Sao Tome and Principe	Secondary Education	1985	3.018
Country	Education	Cohort	TFR
Sao Tome and Principe	Secondary Education	1990	3.080
Sao Tome and Principe	Secondary Education	1995	2.196

Sao Tome and Principe	Secondary Education	2000	2.199
Sao Tome and Principe	Higher Education	1985	2.327
Sao Tome and Principe	Higher Education	1990	2.375
Sao Tome and Principe	Higher Education	1995	1.689
Sao Tome and Principe	Higher Education	2000	1.692
Senegal	No Education	1985	5.299
Senegal	No Education	1990	5.248
Senegal	No Education	1995	5.019
Senegal	No Education	2000	4.604
Senegal	Secondary Education	1985	3.629
Senegal	Secondary Education	1990	3.589
Senegal	Secondary Education	1995	3.412
Senegal	Secondary Education	2000	3.092
Senegal	Higher Education	1985	2.939
Senegal	Higher Education	1990	2.907
Senegal	Higher Education	1995	2.763
Senegal	Higher Education	2000	2.502
Sierra Leone	No Education	1985	5.057
Sierra Leone	No Education	1990	4.816
Sierra Leone	No Education	1995	4.913
Sierra Leone	No Education	2000	4.641
Sierra Leone	Secondary Education	1985	3.338
Sierra Leone	Secondary Education	1990	3.158
Sierra Leone	Secondary Education	1995	3.230
Sierra Leone	Secondary Education	2000	3.027
Sierra Leone	Higher Education	1985	2.951
Sierra Leone	Higher Education	1990	2.790
Sierra Leone	Higher Education	1995	2.854
Sierra Leone	Higher Education	2000	2.674
South Africa	No Education	1985	3.576
South Africa	No Education	1990	3.365
South Africa	No Education	1995	3.171
South Africa	No Education	2000	2.969
South Africa	Secondary Education	1985	2.157
South Africa	Secondary Education	1990	2.013
South Africa	Secondary Education	1995	1.881
South Africa	Secondary Education	2000	1.746
South Africa	Higher Education	1985	1.934
Country	Education	Cohort	TFR
South Africa	Higher Education	1990	1.804
South Africa	Higher Education	1995	1.685

South Africa	Higher Education	2000	1.563
Sudan	No Education	1985	5.312
Sudan	No Education	1990	4.859
Sudan	No Education	1995	4.421
Sudan	No Education	2000	3.977
Sudan	Secondary Education	1985	4.304
Sudan	Secondary Education	1990	3.887
Sudan	Secondary Education	1995	3.490
Sudan	Secondary Education	2000	3.092
Sudan	Higher Education	1985	3.883
Sudan	Higher Education	1990	3.505
Sudan	Higher Education	1995	3.145
Sudan	Higher Education	2000	2.784
Togo	No Education	1985	5.038
Togo	No Education	1990	4.763
Togo	No Education	1995	4.405
Togo	No Education	2000	4.121
Togo	Secondary Education	1985	3.654
Togo	Secondary Education	1990	3.427
Togo	Secondary Education	1995	3.135
Togo	Secondary Education	2000	2.907
Togo	Higher Education	1985	2.906
Togo	Higher Education	1990	2.724
Togo	Higher Education	1995	2.491
Togo	Higher Education	2000	2.308
Tunisia	No Education	1985	4.205
Tunisia	No Education	1990	3.785
Tunisia	No Education	1995	3.515
Tunisia	No Education	2000	3.232
Tunisia	Secondary Education	1985	1.662
Tunisia	Secondary Education	1990	1.474
Tunisia	Secondary Education	1995	1.355
Tunisia	Secondary Education	2000	1.231
Tunisia	Higher Education	1985	2.336
Tunisia	Higher Education	1990	2.070
Tunisia	Higher Education	1995	1.902
Tunisia	Higher Education	2000	1.727
Uganda	No Education	1985	6.800
Country	Education	Cohort	TFR
Uganda	No Education	1990	6.516
Uganda	No Education	1995	6.211

Uganda	No Education	2000	6.061
Uganda	Secondary Education	1985	4.370
Uganda	Secondary Education	1990	4.163
Uganda	Secondary Education	1995	3.942
Uganda	Secondary Education	2000	3.833
Uganda	Higher Education	1985	3.522
Uganda	Higher Education	1990	3.354
Uganda	Higher Education	1995	3.175
Uganda	Higher Education	2000	3.087
Zambia	No Education	1985	6.489
Zambia	No Education	1990	6.156
Zambia	No Education	1995	5.760
Zambia	No Education	2000	5.522
Zambia	Secondary Education	1985	4.241
Zambia	Secondary Education	1990	3.994
Zambia	Secondary Education	1995	3.702
Zambia	Secondary Education	2000	3.529
Zambia	Higher Education	1985	3.314
Zambia	Higher Education	1990	3.120
Zambia	Higher Education	1995	2.891
Zambia	Higher Education	2000	2.755
Zimbabwe	No Education	1985	4.940
Zimbabwe	No Education	1990	4.562
Zimbabwe	No Education	1995	4.371
Zimbabwe	No Education	2000	4.211
Zimbabwe	Secondary Education	1985	3.474
Zimbabwe	Secondary Education	1990	3.173
Zimbabwe	Secondary Education	1995	3.022
Zimbabwe	Secondary Education	2000	2.896
Zimbabwe	Higher Education	1985	2.716
Zimbabwe	Higher Education	1990	2.479
Zimbabwe	Higher Education	1995	2.360
Zimbabwe	Higher Education	2000	2.261
Angola	Diff_edu	1985	5.867
Angola	Diff_edu	1990	5.692
Angola	Diff_edu	1995	5.077
Angola	Diff_edu	2000	4.485
Benin	Diff_edu	1985	5.220
Country	Education	Cohort	TFR
Benin	Diff_edu	1990	4.727
Benin	Diff_edu	1995	4.113

Benin	Diff_edu	2000	4.187
Burkina Faso	Diff_edu	1985	5.201
Burkina Faso	Diff_edu	1990	5.124
Burkina Faso	Diff_edu	1995	4.821
Burkina Faso	Diff_edu	2000	4.636
Burundi	Diff_edu	1985	5.449
Burundi	Diff_edu	1990	5.282
Burundi	Diff_edu	1995	4.382
Burundi	Diff_edu	2000	3.930
Cameroon	Diff_edu	1985	4.904
Cameroon	Diff_edu	1990	4.111
Cameroon	Diff_edu	1995	3.669
Cameroon	Diff_edu	2000	3.586
Central African Republic	Diff_edu	1985	5.101
Central African Republic	Diff_edu	1990	4.651
Central African Republic	Diff_edu	1995	4.702
Central African Republic	Diff_edu	2000	4.479
Chad	Diff_edu	1985	6.791
Chad	Diff_edu	1990	6.793
Chad	Diff_edu	1995	5.897
Chad	Diff_edu	2000	5.945
Comoros	Diff_edu	1985	4.752
Comoros	Diff_edu	1990	4.403
Comoros	Diff_edu	1995	3.512
Comoros	Diff_edu	2000	3.794
Congo	Diff_edu	1985	4.465
Congo	Diff_edu	1990	3.415
Congo	Diff_edu	1995	3.123
Congo	Diff_edu	2000	3.411
Egypt	Diff_edu	1985	3.030
Egypt	Diff_edu	1990	2.498
Egypt	Diff_edu	1995	2.045
Egypt	Diff_edu	2000	1.655
Ethiopia	Diff_edu	1985	4.407
Ethiopia	Diff_edu	1990	4.054
Ethiopia	Diff_edu	1995	3.734
Ethiopia	Diff_edu	2000	3.522
Gabon	Diff_edu	1985	3.930
Country	Education	Cohort	TFR
Gabon	Diff_edu	1990	3.606
Gabon	Diff_edu	1995	2.488

Gabon	Diff_edu	2000	2.691
Gambia	Diff_edu	1985	5.370
Gambia	Diff_edu	1990	4.758
Gambia	Diff_edu	1995	4.284
Gambia	Diff_edu	2000	3.723
Ghana	Diff_edu	1985	3.937
Ghana	Diff_edu	1990	3.838
Ghana	Diff_edu	1995	3.667
Ghana	Diff_edu	2000	3.516
Guinea	Diff_edu	1985	4.752
Guinea	Diff_edu	1990	4.586
Guinea	Diff_edu	1995	4.146
Guinea	Diff_edu	2000	4.057
Kenya	Diff_edu	1985	3.878
Kenya	Diff_edu	1990	3.664
Kenya	Diff_edu	1995	3.472
Kenya	Diff_edu	2000	3.140
Lesotho	Diff_edu	1985	3.019
Lesotho	Diff_edu	1990	2.489
Lesotho	Diff_edu	1995	2.272
Lesotho	Diff_edu	2000	2.097
Liberia	Diff_edu	1985	4.235
Liberia	Diff_edu	1990	3.943
Liberia	Diff_edu	1995	3.947
Liberia	Diff_edu	2000	3.397
Madagascar	Diff_edu	1985	4.218
Madagascar	Diff_edu	1990	3.804
Madagascar	Diff_edu	1995	3.602
Madagascar	Diff_edu	2000	3.403
Malawi	Diff_edu	1985	4.416
Malawi	Diff_edu	1990	3.982
Malawi	Diff_edu	1995	3.630
Malawi	Diff_edu	2000	3.355
Mali	Diff_edu	1985	6.260
Mali	Diff_edu	1990	6.052
Mali	Diff_edu	1995	5.820
Mali	Diff_edu	2000	5.666
Morocco	Diff_edu	1985	2.580
Country	Education	Cohort	TFR
Morocco	Diff_edu	1990	2.424
Morocco	Diff_edu	1995	1.952

Morocco	Diff_edu	2000	1.681
Mozambique	Diff_edu	1985	4.959
Mozambique	Diff_edu	1990	4.459
Mozambique	Diff_edu	1995	4.062
Mozambique	Diff_edu	2000	3.857
Namibia	Diff_edu	1985	3.470
Namibia	Diff_edu	1990	3.232
Namibia	Diff_edu	1995	3.275
Namibia	Diff_edu	2000	2.661
Niger	Diff_edu	1985	6.895
Niger	Diff_edu	1990	6.720
Niger	Diff_edu	1995	6.249
Niger	Diff_edu	2000	5.952
Nigeria	Diff_edu	1985	5.547
Nigeria	Diff_edu	1990	4.938
Nigeria	Diff_edu	1995	4.582
Nigeria	Diff_edu	2000	4.047
Rwanda	Diff_edu	1985	3.949
Rwanda	Diff_edu	1990	3.578
Rwanda	Diff_edu	1995	3.139
Rwanda	Diff_edu	2000	2.990
Sao Tome and Principe	Diff_edu	1985	4.494
Sao Tome and Principe	Diff_edu	1990	4.412
Sao Tome and Principe	Diff_edu	1995	3.106
Sao Tome and Principe	Diff_edu	2000	3.041
Senegal	Diff_edu	1985	4.734
Senegal	Diff_edu	1990	4.590
Senegal	Diff_edu	1995	4.280
Senegal	Diff_edu	2000	3.815
Sierra Leone	Diff_edu	1985	4.695
Sierra Leone	Diff_edu	1990	4.357
Sierra Leone	Diff_edu	1995	4.340
Sierra Leone	Diff_edu	2000	4.006
South Africa	Diff_edu	1985	2.296
South Africa	Diff_edu	1990	2.108
South Africa	Diff_edu	1995	1.938
South Africa	Diff_edu	2000	1.769
Sudan	Diff_edu	1985	5.090
Country	Education	Cohort	TFR
Sudan	Diff_edu	1990	4.503
Sudan	Diff_edu	1995	3.948

Sudan	Diff_edu	2000	3.407
Togo	Diff_edu	1985	4.584
Togo	Diff_edu	1990	4.217
Togo	Diff_edu	1995	3.764
Togo	Diff_edu	2000	3.431
Tunisia	Diff_edu	1985	2.376
Tunisia	Diff_edu	1990	1.845
Tunisia	Diff_edu	1995	1.701
Tunisia	Diff_edu	2000	1.558
Uganda	Diff_edu	1985	5.238
Uganda	Diff_edu	1990	4.937
Uganda	Diff_edu	1995	4.622
Uganda	Diff_edu	2000	4.441
Zambia	Diff_edu	1985	4.993
Zambia	Diff_edu	1990	4.572
Zambia	Diff_edu	1995	4.113
Zambia	Diff_edu	2000	3.796
Zimbabwe	Diff_edu	1985	3.725
Zimbabwe	Diff_edu	1990	3.333
Zimbabwe	Diff_edu	1995	3.105
Zimbabwe	Diff_edu	2000	2.908
Angola	Diff_edu	1955	6.950
Angola	Diff_edu	1960	6.680
Angola	Diff_edu	1965	6.503
Angola	Diff_edu	1970	6.372
Angola	Diff_edu	1975	6.270
Angola	Diff_edu	1980	6.109
Angola	Diff_edu	1985	5.867
Benin	Diff_edu	1955	6.640
Benin	Diff_edu	1960	6.271
Benin	Diff_edu	1965	5.863
Benin	Diff_edu	1970	5.549
Benin	Diff_edu	1975	5.462
Benin	Diff_edu	1980	5.367
Benin	Diff_edu	1985	5.220
Burkina Faso	Diff_edu	1955	6.900
Burkina Faso	Diff_edu	1960	6.603
Burkina Faso	Diff_edu	1965	6.417
Country	Education	Cohort	TFR
Burkina Faso	Diff_edu	1970	6.126
Burkina Faso	Diff_edu	1975	5.829

Burkina Faso	Diff_edu	1980	5.523
Burkina Faso	Diff_edu	1985	5.201
Burundi	Diff_edu	1955	7.068
Burundi	Diff_edu	1960	7.042
Burundi	Diff_edu	1965	6.799
Burundi	Diff_edu	1970	6.546
Burundi	Diff_edu	1975	6.162
Burundi	Diff_edu	1980	5.751
Burundi	Diff_edu	1985	5.480
Cameroon	Diff_edu	1955	6.260
Cameroon	Diff_edu	1960	6.076
Cameroon	Diff_edu	1965	5.869
Cameroon	Diff_edu	1970	5.572
Cameroon	Diff_edu	1975	5.330
Cameroon	Diff_edu	1980	5.220
Cameroon	Diff_edu	1985	4.904
Central African Republic	Diff_edu	1955	5.929
Central African Republic	Diff_edu	1960	5.894
Central African Republic	Diff_edu	1965	5.917
Central African Republic	Diff_edu	1970	5.907
Central African Republic	Diff_edu	1975	5.757
Central African Republic	Diff_edu	1980	5.360
Central African Republic	Diff_edu	1985	5.101
Chad	Diff_edu	1955	6.791
Chad	Diff_edu	1960	7.084
Chad	Diff_edu	1965	7.393
Chad	Diff_edu	1970	7.370
Chad	Diff_edu	1975	6.994
Chad	Diff_edu	1980	6.809
Chad	Diff_edu	1985	6.791
Comoros	Diff_edu	1955	6.607
Comoros	Diff_edu	1960	6.169
Comoros	Diff_edu	1965	5.541
Comoros	Diff_edu	1970	5.030
Comoros	Diff_edu	1975	4.816
Comoros	Diff_edu	1980	4.580
Comoros	Diff_edu	1985	4.752
Congo	Diff_edu	1955	5.535
Country	Education	Cohort	TFR
Congo	Diff_edu	1960	5.257
Congo	Diff_edu	1965	4.987

Congo	Diff_edu	1970	4.748
Congo	Diff_edu	1975	4.597
Congo	Diff_edu	1980	4.528
Congo	Diff_edu	1985	4.465
Egypt	Diff_edu	1955	5.026
Egypt	Diff_edu	1960	4.519
Egypt	Diff_edu	1965	4.093
Egypt	Diff_edu	1970	3.722
Egypt	Diff_edu	1975	3.383
Egypt	Diff_edu	1980	3.163
Egypt	Diff_edu	1985	3.030
Ethiopia	Diff_edu	1955	6.653
Ethiopia	Diff_edu	1960	6.587
Ethiopia	Diff_edu	1965	6.423
Ethiopia	Diff_edu	1970	6.053
Ethiopia	Diff_edu	1975	5.678
Ethiopia	Diff_edu	1980	5.058
Ethiopia	Diff_edu	1985	4.407
Gabon	Diff_edu	1955	5.395
Gabon	Diff_edu	1960	5.160
Gabon	Diff_edu	1965	4.932
Gabon	Diff_edu	1970	4.593
Gabon	Diff_edu	1975	4.310
Gabon	Diff_edu	1980	4.063
Gabon	Diff_edu	1985	3.930
Gambia	Diff_edu	1955	6.496
Gambia	Diff_edu	1960	6.413
Gambia	Diff_edu	1965	6.234
Gambia	Diff_edu	1970	5.946
Gambia	Diff_edu	1975	5.772
Gambia	Diff_edu	1980	5.675
Gambia	Diff_edu	1985	5.370
Ghana	Diff_edu	1955	5.714
Ghana	Diff_edu	1960	5.341
Ghana	Diff_edu	1965	4.999
Ghana	Diff_edu	1970	4.664
Ghana	Diff_edu	1975	4.398
Ghana	Diff_edu	1980	4.169
Country	Education	Cohort	TFR
Ghana	Diff_edu	1985	3.937
Guinea	Diff_edu	1955	6.195

Guinea	Diff_edu	1960	6.165
Guinea	Diff_edu	1965	6.047
Guinea	Diff_edu	1970	5.725
Guinea	Diff_edu	1975	5.374
Guinea	Diff_edu	1980	5.145
Guinea	Diff_edu	1985	4.752
Kenya	Diff_edu	1955	6.512
Kenya	Diff_edu	1960	6.051
Kenya	Diff_edu	1965	5.494
Kenya	Diff_edu	1970	4.968
Kenya	Diff_edu	1975	4.515
Kenya	Diff_edu	1980	4.096
Kenya	Diff_edu	1985	3.878
Lesotho	Diff_edu	1955	4.939
Lesotho	Diff_edu	1960	4.642
Lesotho	Diff_edu	1965	4.163
Lesotho	Diff_edu	1970	3.701
Lesotho	Diff_edu	1975	3.453
Lesotho	Diff_edu	1980	3.133
Lesotho	Diff_edu	1985	3.019
Liberia	Diff_edu	1955	6.352
Liberia	Diff_edu	1960	6.184
Liberia	Diff_edu	1965	5.972
Liberia	Diff_edu	1970	5.571
Liberia	Diff_edu	1975	5.195
Liberia	Diff_edu	1980	4.880
Liberia	Diff_edu	1985	4.235
Madagascar	Diff_edu	1955	6.407
Madagascar	Diff_edu	1960	5.867
Madagascar	Diff_edu	1965	5.530
Madagascar	Diff_edu	1970	5.079
Madagascar	Diff_edu	1975	4.864
Madagascar	Diff_edu	1980	4.591
Madagascar	Diff_edu	1985	4.218
Malawi	Diff_edu	1955	6.694
Malawi	Diff_edu	1960	6.310
Malawi	Diff_edu	1965	6.004
Malawi	Diff_edu	1970	5.733
Country	Education	Cohort	TFR
Malawi	Diff_edu	1975	5.378
Malawi	Diff_edu	1980	4.869

Malawi	Diff_edu	1985	4.416
Mali	Diff_edu	1955	7.135
Mali	Diff_edu	1960	6.926
Mali	Diff_edu	1965	6.743
Mali	Diff_edu	1970	6.512
Mali	Diff_edu	1975	6.328
Mali	Diff_edu	1980	6.254
Mali	Diff_edu	1985	6.260
Morocco	Diff_edu	1955	4.832
Morocco	Diff_edu	1960	4.088
Morocco	Diff_edu	1965	3.395
Morocco	Diff_edu	1970	2.985
Morocco	Diff_edu	1975	2.774
Morocco	Diff_edu	1980	2.673
Morocco	Diff_edu	1985	2.580
Mozambique	Diff_edu	1955	6.044
Mozambique	Diff_edu	1960	5.931
Mozambique	Diff_edu	1965	5.608
Mozambique	Diff_edu	1970	5.490
Mozambique	Diff_edu	1975	5.297
Mozambique	Diff_edu	1980	5.127
Mozambique	Diff_edu	1985	4.959
Namibia	Diff_edu	1955	5.418
Namibia	Diff_edu	1960	4.851
Namibia	Diff_edu	1965	4.385
Namibia	Diff_edu	1970	3.929
Namibia	Diff_edu	1975	3.678
Namibia	Diff_edu	1980	3.523
Namibia	Diff_edu	1985	3.470
Niger	Diff_edu	1955	7.375
Niger	Diff_edu	1960	7.538
Niger	Diff_edu	1965	7.576
Niger	Diff_edu	1970	7.383
Niger	Diff_edu	1975	7.169
Niger	Diff_edu	1980	7.037
Niger	Diff_edu	1985	6.895
Nigeria	Diff_edu	1955	6.436
Nigeria	Diff_edu	1960	6.511
Country	Education	Cohort	TFR
Nigeria	Diff_edu	1965	6.252
Nigeria	Diff_edu	1970	6.039

Nigeria	Diff_edu	1975	5.825
Nigeria	Diff_edu	1980	5.620
Nigeria	Diff_edu	1985	5.547
Rwanda	Diff_edu	1955	7.058
Rwanda	Diff_edu	1960	6.491
Rwanda	Diff_edu	1965	5.978
Rwanda	Diff_edu	1970	5.436
Rwanda	Diff_edu	1975	5.110
Rwanda	Diff_edu	1980	4.583
Rwanda	Diff_edu	1985	3.949
Sao Tome and Principe	Diff_edu	1955	6.851
Sao Tome and Principe	Diff_edu	1960	6.292
Sao Tome and Principe	Diff_edu	1965	6.023
Sao Tome and Principe	Diff_edu	1970	5.590
Sao Tome and Principe	Diff_edu	1975	5.017
Sao Tome and Principe	Diff_edu	1980	4.651
Sao Tome and Principe	Diff_edu	1985	4.494
Senegal	Diff_edu	1955	6.750
Senegal	Diff_edu	1960	6.314
Senegal	Diff_edu	1965	5.910
Senegal	Diff_edu	1970	5.487
Senegal	Diff_edu	1975	5.125
Senegal	Diff_edu	1980	4.878
Senegal	Diff_edu	1985	4.734
Sierra Leone	Diff_edu	1955	6.662
Sierra Leone	Diff_edu	1960	6.431
Sierra Leone	Diff_edu	1965	6.130
Sierra Leone	Diff_edu	1970	5.758
Sierra Leone	Diff_edu	1975	5.341
Sierra Leone	Diff_edu	1980	4.994
Sierra Leone	Diff_edu	1985	4.695
South Africa	Diff_edu	1955	4.041
South Africa	Diff_edu	1960	3.703
South Africa	Diff_edu	1965	3.287
South Africa	Diff_edu	1970	2.846
South Africa	Diff_edu	1975	2.526
South Africa	Diff_edu	1980	2.365
South Africa	Diff_edu	1985	2.296
Country	Education	Cohort	TFR
Sudan	Diff_edu	1955	6.646
Sudan	Diff_edu	1960	6.057

Sudan	Diff_edu	1965	5.717
Sudan	Diff_edu	1970	5.295
Sudan	Diff_edu	1975	4.975
Sudan	Diff_edu	1980	4.838
Sudan	Diff_edu	1985	5.090
Togo	Diff_edu	1955	6.107
Togo	Diff_edu	1960	5.782
Togo	Diff_edu	1965	5.488
Togo	Diff_edu	1970	5.195
Togo	Diff_edu	1975	4.844
Togo	Diff_edu	1980	4.662
Togo	Diff_edu	1985	4.584
Tunisia	Diff_edu	1955	4.387
Tunisia	Diff_edu	1960	3.668
Tunisia	Diff_edu	1965	3.159
Tunisia	Diff_edu	1970	2.624
Tunisia	Diff_edu	1975	2.465
Tunisia	Diff_edu	1980	2.270
Tunisia	Diff_edu	1985	2.376
Uganda	Diff_edu	1955	6.928
Uganda	Diff_edu	1960	6.857
Uganda	Diff_edu	1965	6.699
Uganda	Diff_edu	1970	6.484
Uganda	Diff_edu	1975	6.296
Uganda	Diff_edu	1980	5.773
Uganda	Diff_edu	1985	5.238
Zambia	Diff_edu	1955	6.648
Zambia	Diff_edu	1960	6.319
Zambia	Diff_edu	1965	6.053
Zambia	Diff_edu	1970	5.787
Zambia	Diff_edu	1975	5.505
Zambia	Diff_edu	1980	5.141
Zambia	Diff_edu	1985	4.993
Zimbabwe	Diff_edu	1955	5.599
Zimbabwe	Diff_edu	1960	5.004
Zimbabwe	Diff_edu	1965	4.338
Zimbabwe	Diff_edu	1970	3.832
Zimbabwe	Diff_edu	1975	3.673
Country	Education	Cohort	TFR
Zimbabwe	Diff_edu	1980	3.605
Zimbabwe	Diff_edu	1985	3.725

SI References

1. Rutstein SO, Rojas G. Guide to DHS statistics. Calverton MD ORC Macro. 2006;38:78.
2. Durowaa-Boateng A, Yildiz D, Goujon A. A Bayesian model for the reconstruction of education-and age-specific fertility rates. Demogr Res. 2023;49:809–48.
3. Lee RD, Carter LR. Modeling and Forecasting U.S. Mortality. J Am Stat Assoc. 1992 Sep;87(419):659–71.
4. Li N, Wu Z. Forecasting cohort incomplete fertility: A method and an application. Popul Stud. 2003 Nov;57(3):303–20.
5. Lutz W, Reiter C, Özdemir C, Yildiz D, Guimaraes R, Goujon A. Skills-adjusted human capital shows rising global gap. Proc Natl Acad Sci. 2021 Feb 16;118(7):e2015826118.
